# Strain engineering in perovskite solar cells and its impacts on carrier dynamics

**DOI:** 10.1038/s41467-019-08507-4

**Published:** 2019-02-18

**Authors:** Cheng Zhu, Xiuxiu Niu, Yuhao Fu, Nengxu Li, Chen Hu, Yihua Chen, Xin He, Guangren Na, Pengfei Liu, Huachao Zai, Yang Ge, Yue Lu, Xiaoxing Ke, Yang Bai, Shihe Yang, Pengwan Chen, Yujing Li, Manling Sui, Lijun Zhang, Huanping Zhou, Qi Chen

**Affiliations:** 1 Beijing Key Laboratory of Construction Tailorable Advanced Functional Materials and Green Applications, Advanced Materials Experimental Center, School of Materials Science & Engineering, Beijing Institute of Technology, 100081 Beijing, China; 2 State Key Laboratory of Superhard Materials, Key Laboratory of Automobile Materials of MOE, and School of Materials Science and Engineering, Jilin University, 130012 Changchun, China; 3 Department of Materials Science and Engineering, College of Engineering, Peking University, 100871 Beijing, China; 4 Department of Chemistry, The Hong Kong University of Science and Technology, Clear Water Bay, Kowloon, Hong Kong China; 5 Department of Materials Science and Engineering, College of Science, China University of Petroleum, 102249 Beijing, China; 6 Institute of Microstructure and Properties of Advanced Materials, Beijing University of Technology, 100124 Beijing, China; 7 Guangdong Key Lab of Nano-Micro Material Research, School of Chemical Biology and Biotechnology, Shenzhen Graduate School, Peking University, Xili University Town, 518055 Shenzhen, Guangdong China; 8 State Key Laboratory of Explosion Science and Technology, Beijing Institute of Technology, 100081 Beijing, China

## Abstract

The mixed halide perovskites have emerged as outstanding light absorbers for efficient solar cells. Unfortunately, it reveals inhomogeneity in these polycrystalline films due to composition separation, which leads to local lattice mismatches and emergent residual strains consequently. Thus far, the understanding of these residual strains and their effects on photovoltaic device performance is absent. Herein we study the evolution of residual strain over the films by depth-dependent grazing incident X-ray diffraction measurements. We identify the gradient distribution of in-plane strain component perpendicular to the substrate. Moreover, we reveal its impacts on the carrier dynamics over corresponding solar cells, which is stemmed from the strain induced energy bands bending of the perovskite absorber as indicated by first-principles calculations. Eventually, we modulate the status of residual strains in a controllable manner, which leads to enhanced PCEs up to 20.7% (certified) in devices via rational strain engineering.

## Abstract (web summary)

The residual strains in the mixed halide perovskite thin films and their effects on the solar cell devices are less understood. Here Zhu et al. study the impact of the gradient in-plane strain on the carrier dynamics of the strained perovskite films and optimize the device efficiency.

## Introduction

Over the past decade, hybrid organic−inorganic halide perovskites have received enormous interest as low-cost and highly efficient light absorbers in photovoltaics^1–6^. The power conversion efficiency (PCE) of perovskite solar cells has shortly surpassed 22% in the lab scale^7^, and that of larger-area (>1 cm^2^) devices has exceeded 20%^8–11^. The highest PCEs are mostly achieved by employing the mixed halide perovskites (e.g., (HC(NH_2_)_2_PbI_3_)_0.85_(CH_3_NH_3_PbBr_3_)_0.15_ i.e., (FAPbI_3_)_0.85_(MAPbBr_3_)_0.15_). Through element substitution, it provides a largely unexplored compositional space to tailor the physiochemical properties of corresponding materials for efficient and stable devices^1,12–15^. However, the mixed hybrid perovskites potentially suffer from materials inhomogeneity partially due to composition separation^16,17^ and/or thermal stress. This may be originated from substantial chemical mismatch among each component, and the nonequilibrium growth conditions during film fabrication. On one hand, serious material inhomogeneity is regarded as phase separation, deviating from the originally desired materials properties to deteriorate the resultant device performance (both efficiency and operational stability)^18,19^. On the other hand, moderate material inhomogeneity correlates to local lattice mismatches and emergent residual strains in perovskite films. It deserves more attention, because such residual strains should result in lattice distortion of microscopic crystal structure, and further affect optoelectronic properties of the perovskite thin film^20–25^.

Recently, the residual strains have been identified in the single-composition MAPbI_3_ polycrystalline films, and were found to influence stability of perovskite films under illumination^22^. In an even wider spectrum of semiconductors, strain has been extensively investigated, and accordingly, strain engineering has been exploited to tailor the optoelectronic functionalities. For instance, the application of tensile strain was found to improve light emission in Germanium crystals and drive indirect-to-direct optical transition in bilayered two-dimensional WSe_2_^26–28^. Strain compensation strategy has been demonstrated to improve efficiencies of GaAs-based quantum dot solar cells^29,30^. Strain-engineered MoS_2_ monolayer was also proposed to have broad-range capture of solar energy^26^. For hybrid halide perovskites, especially mixed perovskites, in-depth understanding of their characteristics and effects on the material’s optoelectronic properties, is still absent. This precludes effective employment of strain engineering to further enhance the device performance.

In this report, we probe the residual strain distribution profiles in the mixed perovskite thin films and its effects on photovoltaic device efficiency. We investigate the evolution of in-plane residual strain over the film thickness in the typical mixed perovskite (FAPbI_3_)_0.85_(MAPbBr_3_)_0.15_ by using grazing incident X-ray diffraction (GIXRD) measurement. We identify a gradient distribution of in-plane strain component that correlates to the composition inhomogeneity perpendicular to the substrate. We further demonstrate a feasible method to modulate the tensile or compressive nature of residual strain and even its gradient over perovskite films in a controllable manner. With the aid of first-principles calculations, we find that the strain gradient induces energy bands bending and thus affects the carrier dynamics across the interfaces over the solar cell. By deliberately engineering the residual strains to enhance carrier extraction efficiency, we successfully fabricate strain-engineered perovskite solar cells to achieve enhanced PCEs up to 20.7% (certified).

## Results

### Probing residual strain gradient of mixed perovskite films

**Fig. 1:**
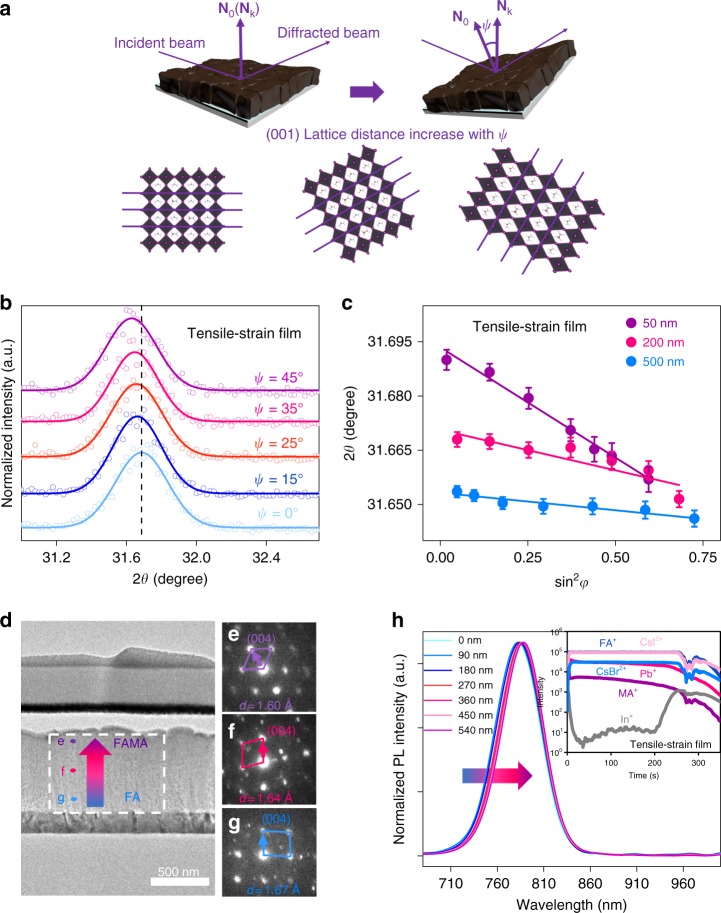
Gradient lattice structure characterization. **a** Schematic illustration of the residual strain distribution measurement. The corresponding XRD patterns and lattice structure strain information can be obtained by fixing the test crystal plane and adjusting the instrument tilt angle *ψ*, where **N**_0_ is the sample normal direction and **N**_k_ is the diffraction vector. **b** GIXRD spectrum at different tilt angles at the depth of 50 nm for the tensile-strained film. **c** Residual strain distribution in the depth of 50, 200, 500 nm for the tensile-strained film (measured (points) and Gauss fitted (line) diffraction strain data as a function of sin^2^*φ*). The error bar indicates standard deviation of the 2*θ*. **d** The cross-sectional TEM image of device. **e**, **f**, **g** The nano-beam electron diffraction patterns ([100] zone axis and TEM specimens is FIBed), corresponding with e-f-g point in **d**, confirming the FAMA hybrid perovskite phase structure transform to nearly pure FA phase from the surface to the bottom of perovskite film according to the larger quadrangle. **h** PL depth profile of confocal fluorescence microscope, the inset represents TOF-SIMS depth profiles of the (FAPbI_3_)_0.85_(MAPbBr_3_)_0.15_ perovskite film with tensile strain. XRD X-ray diffraction, TEM transmission electron microscopy

In the direction parallel to substrates, the grain-to-grain inhomogeneity in polycrystalline films was already observed by adopting photoluminescence in a scanning electron microscopy (PL-SEM) and conductive-atomic force microscopy (C-AFM)^31^. However, it lacks depth profile along the film thickness regarding the lattice structure inhomogeneity. At the macroscopic level, the vertical homogeneity of thin films can be quantitatively evaluated by residual strain to reflect the lattice mismatch^20,22,32^. The macroscopic residual strain is an internal strain in polycrystalline materials that is balanced over a wide range of grains. We explored the residual strain distribution for mixed perovskite films vertically with the depth-dependent GIXRD measurement, wherein the classical sin^2^*φ* measurement is combined with grazing incident X-ray diffraction to probe the in-plane residual strain. This method has been used in thin films of ZrO_2_ and TiN, which provides reliable depth resolution to reveal lattice structure evolution^33–35^. As depicted in Fig. 1a, we fixed the 2*θ* and varied the instrument tilt angle *ψ* to obtain corresponding X-ray diffraction (XRD) patterns as shown in Fig. 1b. For detailed test information, please see the Supplementary method, Supplementary Fig. 1 and Supplementary Table 1. The mixed perovskite films on the SnO_2_/ITO/glass substrates showed XRD peaks (2*θ*) at around 14.0°, 31.6°, and 40.5°, corresponding to (001), (012), and (022) crystallographic planes, respectively^36^. Among all three planes with intensive diffraction peaks, the (012) plane was chosen for further analysis due to its high diffraction angle and multiplicative factor, which provides the most reliable structure symmetry information.

We picked up three representative depths of 50, 200 and 500 nm to check the residual strain in the mixed perovskite films with the nominal formula of (FAPbI_3_)_0.85_(MAPbBr_3_)_0.15_. At each fixed depth, diffraction data were fitted with Gaussian distribution function and it clearly observed a systematic shift in peak position to lower 2*θ* when the penetrated depth increased (Fig. 1b and Supplementary Fig. 2). Generally, sin^2^*φ* and 2*θ* follow a linear relationship and the slope of the fitting line stands for the magnitude of the residual strain (see equation (1) in Supplementary method). As shown in Fig. 1b, all fitting lines exhibited negative value in the slopes, which means (012) crystal planes exhibit enlarged distance when turning to the out-of-plane direction at the same detected depth. It thus indicates the entire sample is subjected to tensile strain. It is in agreement with previous study wherein the residual tensile strain was observed in perovskite thin films upon the similar annealing process^22^.

With the increase of probe depth, the fitting lines show smaller slopes in absolute value, which implies the macroscopic residual tensile strain gradually decreases. Moreover, we found the most significant deviation in lattice constant at 50 nm as compared to that of 200 and 500 nm. Besides, the (001) and (022) crystal planes were also found to follow the similar trend (Supplementary Fig. 2). It indicates the residual strain inhomogeneity in the mixed perovskite thin film, wherein the top surface of the film bears the largest tensile strain. It is worth noting that residual strain correlates to the lattice distortion, which might affect the carrier dynamics at the relevant interfaces, as will be discussed later. Therefore, the residual tensile strain gradient was clearly identified, which gradually decreases from the top surface to the core in the mixed perovskite polycrystalline thin films.

So far, we have observed the gradient distribution of tensile strain in the perovskite thin film. And it will be interesting to understand the origin of the residual strain. The residual stain is often stemmed from the lattice mismatch due to lattice structure evolution. We thus conducted GIXRD measurements with variable grazing incident angle *ω* (Supplementary Table 1) to reveal the lattice mismatch along the film thickness. These depth-dependent XRD patterns were roughly similar and no new diffraction peaks appeared, indicating the film exhibited the same cubic phase structure at different depths. However, we observed a systematic shift of diffraction peaks along with the probing depths at 50, 200, and 500 nm in the perovskite film. Take (001) planes for example, the corresponding diffraction peaks for 50, 200, and 500 nm exhibited peak centers at 14.01°, 13.94°, and 13.90°, respectively (Supplementary Fig. 3). Among different crystal planes of (001), (012) and (022), corresponding peak positions gradually shift towards the low diffraction angle with the increase of probing depths. According to the Bragg’s law, larger lattice constant was expected in the deeper zone of films. Thus, the crystal structure inhomogeneity along the film thickness is detected unambiguously. To provide the evidence regarding the structure inhomogeneity in the microscopic level, transmission electron microscopy (TEM) nano-beam electron diffraction measurement was further carried out to investigate crystal structure evolution along the depth direction of mixed perovskite films. We obtained the high-resolution TEM image with micro-area diffraction patterns to inspect three typical regions with different depths (Fig. 1d–f). After careful calibration of all measured diffraction patterns (Supplementary Table 2), we found that the crystal plane distance increased with scanning depth. As shown in Fig. 1e–g, lattice parameters of (004) planes were measured to be 1.60, 1.64 and 1.67 Å for three individual areas, respectively, which is consistent with the GIXRD results. Combining the microscopic analysis (TEM) and the long-range structure characterization (GIXRD), we clearly illustrate lattice structure evolution developed vertically in the mixed perovskite polycrystalline thin films, wherein the lattice constant decreases from the surface to the bottom.

The above-mentioned crystal structure inhomogeneity is likely to correlate to the composition evolution in mixed perovskite thin films. It is commonly accepted that FAPbI_3_ exhibits the cubic phase with the lattice constant of 6.357 Å^36^. When MA and/or Br element are incorporated to form the mixed perovskites single crystals, they hold the cubic phase with decreased lattice parameters. We observed smaller lattice constant at the surface of the film, which may partially attribute to higher ratio of MA and/or Br components in the long-range ordered FA based perovskite lattices. To identify the distribution of MA and/or Br components, we resorted to time-of-flight secondary ion mass spectrometry (TOF-SIMS) depth profiles and TEM/EDX mapping for the (FAPbI_3_)_0.85_(MAPbBr_3_)_0.15_ perovskite film samples as shown in the inset of Fig. 1h, Supplementary Figs. 4 and  5. It shows a homogeneous distribution of CsBr^2+^, CsI^2+^, FA^+^ in the all samples, which indicates the even distribution of the halogen Br^−^. It is reasonable because the smallest halogen ion Br^−^ can diffuse and be evenly distributed during the crystallization process. To be noted, the signal intensity of MA^+^ fragment decreased substantially from the surface to the core region in all mixed perovskite thin films, consistent with the XRD and TEM results. Unambiguously it reveals the gradient evolution of composition and thus lattice structure across the depth direction due to the gradient distribution of organic cation MA^+^. The non-uniform composition distribution indicates the unique kinetics for film growth, which is possibly related to the coordination strength of different precursors^37^ and film processing conditions. It clearly implies the compositional distribution serve as one major factor that leads to the gradient residual strain.

It is revealed that hybrid perovskites with different lattice structures often exhibit different optoelectronic properties^25,38^ and it is expected to observe the gradient variation in terms of optoelectronic properties within the gradient phase structure of the film. Therefore, we examined the depth-dependent photoluminescence (PL) spectra within the film by using confocal fluorescence microscope. With the increasing depth of the beam probe, a systematic red shift of PL spectra was observed (Fig. 1h and Supplementary Fig. 6). Fitting with the Gaussian distribution function, we found the PL peak positions shifted from 781 to 788 nm, and their full width at half maximum (FWHM) decreased along the film thickness. Since the emission photon energy is determined by the bandgap of semiconductors, it indicates the perovskite film exhibits gradually decreased bandgap from the surface to the bottom vertically. To be noted, it follows the similar trend in gradient evolution of lattice structure and compositional distribution, wherein more MA^+^ ions are incorporated in the perovskite crystals at the top surface of the film. In addition, narrower PL linewidth emission at the deeper region of perovskite film was observed, which may indicate weakened interaction between charge carriers and lattice vibrations (phonons) due to improved film homogeneity and lattice order^39,40^.

### Modulating the residual strains

Based on the analysis above, we reveal that the observed residual strain gradient in perovskite films is closely related to lattice structure evolution due to detectable compositional inhomogeneity. However, it may not be the only contributor that governs the residual strain, given the largest tensile strain concentrated on the film surface. Interestingly, when examining the pure MAPbI_3_ perovskite thin film, we still observed the existence of gradient residual strain (Supplementary Fig. 7). It is thus speculated that the thermal strain may take effects due to the temperature gradient during perovskite film fabrication. To illustrate, the perovskite film is roughly divided into two regions, e.g., the surface and the bottom (near the substrate). When heated on a hot plate, both regions experienced substantial temperature gradient (higher at the bottom). Consequently, the inorganic framework near the bottom expands more in volume upon annealing as compared to that at the surface, which facilitates the insertion of larger cation (FA^+^) to form the perovskite crystalline structure. It possibly results in the inhomogeneous composition in the film, wherein MA^+^ prefers to stay at the surface. Upon cooling, it is reasonable that the surface cools down much faster than that of the core, which leads to less volume shrinkage. As a result, the surface withstands tensile strain and the core is subjected to compressive strain accordingly. Additionally, since the thermal expansion coefficient of perovskites is much larger than that of the substrate, the entire film bears the tensile strain during film cooling process. Thereby, the core experiences not only compressive strain from the surface, but also tensile strain from the substrate, which leads to the gradient residual tensile strain in the perovskite thin film.

**Fig. 2:**
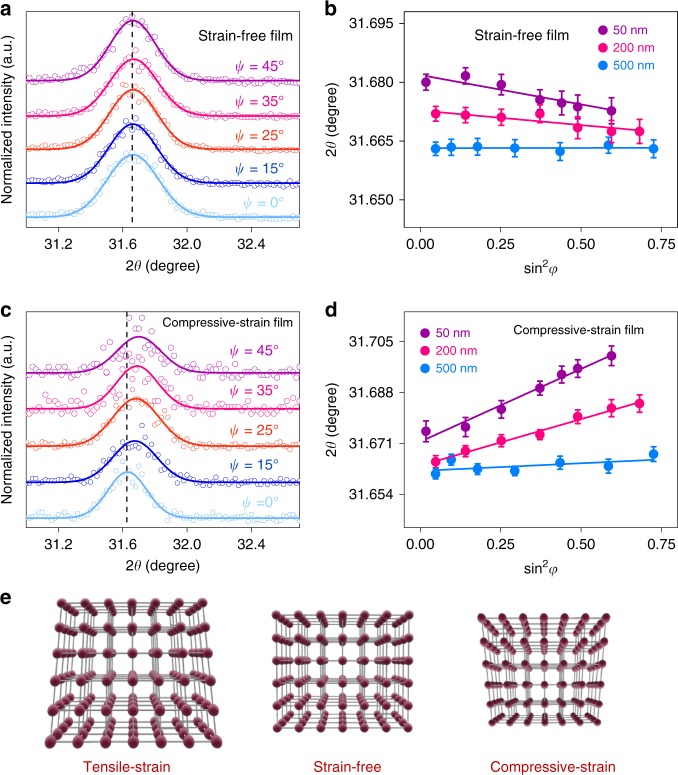
Residual strain distribution measurement with the GIXRD method. **a**, **c** GIXRD spectrum at different tilt angles at the depth of 50 nm for the strain-free film, compressive strained film. **b**, **d** Residual strain distribution in the depth of 50, 200, 500 nm for the strain-free film, compressive strained film (measured (points) and Gauss fitted (line) diffraction strain data as a function of sin^2^*φ*). The error bar indicates standard deviation of the 2*θ*. **e** The schematic representation of the tensile strain state of the film in the top surface, showing the lattice structure with/without tensile strain on the film surface from the perspective of long-range order

To verify the speculation, we attempted to modulate the gradient in-plane residual strain in the perovskite thin films by adjusting the annealing process. We first tried to anneal the film at 150 °C high temperature for a long time, which is a conventional approach to eliminating residual strains in metal alloy materials. Unfortunately, it led to the significant occurrence of PbI_2_ (Supplementary Fig. 7) in the resultant thin films due to the relative poor thermal stability of hybrid perovskites^41^. We then tried to change the temperature gradient when the films were annealed. Simply, we modified the heat treatment process by flipping over the thin film sample to provide an invert temperature gradient. Interestingly, tensile strain was significantly reduced in the samples upon this treatment as shown in Fig. 2a, b. With small strain gradient and the closer lattice distance in the perovskite film, the lattice structure was almost homogeneous. It thus suggests an effective way to significantly reduce the tensile strain inhomogeneity across the film thickness by tuning the temperature gradient during film processing. Furthermore, we tried to directly perform flipped annealing process to the intermediate state film after the spin-coating process ends, which is expected to apply compressive strain with vertical gradient over the film. In contrast to the previous samples, the as-prepared films show the fitting curves possessed slopes in positive values (Fig. 2c, d), which increased along with the probe depth. It clearly indicates films exhibit compressive strain, which is also distributed in a vertical gradient. Unfortunately, we observed a lot of pinholes in the resultant film possibly because the solvent cannot be spread smoothly during the annealing process (Supplementary Fig. 9). Therefore, we demonstrate the temperature gradient is also an important source to residual strain during the film growth, which further suggests a method to manipulate the evolution of lattice structure of polycrystalline thin films and the surface strain.

To investigate whether the upper contacting layer influences the surface strain, we prepared a tensile-strained film to test the surface residual strain as a reference point. Then for the same sample, 1 mL chlorobenzene solution was dripped on the film surface during the spin-coating process and the film was tested. Furthermore, the same sample was coated with Spiro-OMeTAD layer and then tested again. We found the same slope of the fitting curves indicating the magnitude of the surface strain remained constant. (Supplementary Fig. 10) It clearly shows that the chlorobenzene dripped process and/or Spiro-OMeTAD contacting layer does not affect the surface strain significantly.

The residual strain and its gradient distribution reflects the structure inhomogeneity in the perovskite thin film along the vertical direction. It is known that the strain gradient is derived from the XRD peak offset, which actually reveals the variation of lattice parameters leading to the crystal structure mismatch across the thin film. In hybrid halide perovskites, the framework of corner-sharing PbI_6_ octahedral contributes to the electronic configuration, especially at the band-edge^42,43^. It means variations in the inorganic framework would possibly result in the change in optoelectronic properties of the materials. The structure variations include enlargement/shrinkage, tilting, and other deformation of the octahedral network, which can be clearly illustrated by measuring the residual strain. Therefore, it is of great interest to bridge the gulf between the residual strain and the optoelectronic properties of the materials and relevant devices. Given the thin film surface exhibits the most significant strain, it is reasonable focus on the carrier dynamic behavior across the interface via strain modulation, and their effects on device performance.

### Impacts of strain on carrier dynamics and device performance

**Fig. 3:**
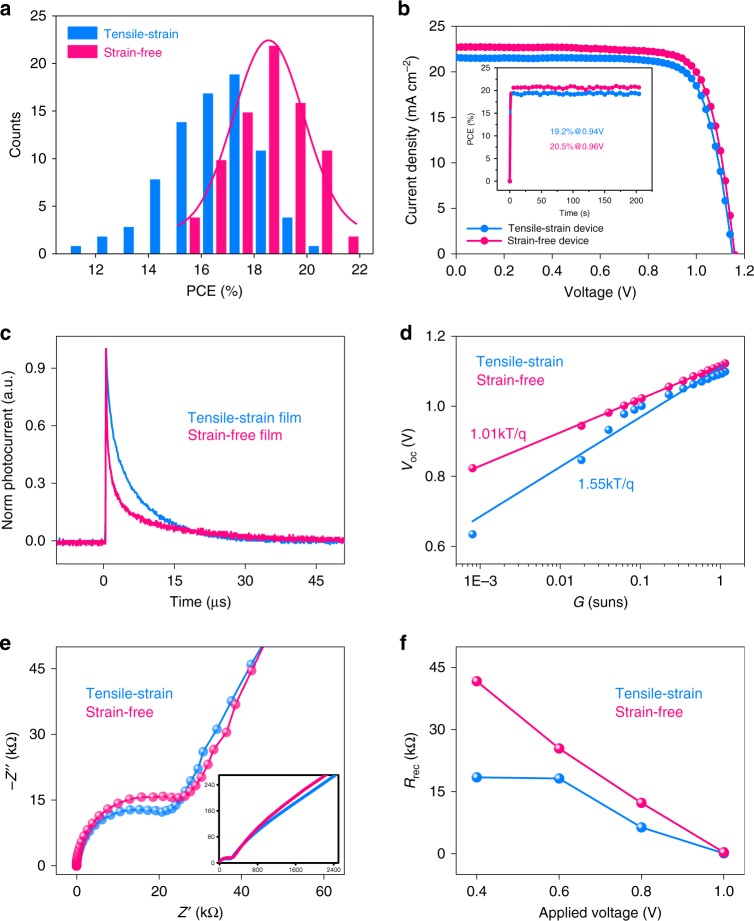
Device performance and carrier dynamic behavior analysis. **a** Histograms of the PCEs for the devices with different strain conditions. **b** *J–V* curves of the tensile strain device and strain-free device. The inset is the stabilized current density measured at a bias voltage (0.94, 0.96 V, respectively). **c** TPC decay curves for PSCs with tensile strain and strain-free conditions. **d** The light-intensity dependence of *V*_OC_ measurement related to tensile strain and strain-free device. **e** EIS curves for PSCs with different strain conditions and the inset is frequency response signal according to frequency parameter from 1 MHz to 100 Hz. **f** Variation of recombination resistance as a function of applied voltage. PCE power conversion efficiency, TPC transient photocurrent, EIS electrochemical impedance spectroscopy

To investigate the impact of the gradient residual strain on the device performance, we first fabricated planar heterojunction solar cells by adopting the perovskite absorbers with/without residual strains. The device architecture follows the regular structure of ITO/SnO_2_/perovskite/Spiro-OMeTAD/Ag. We then compare the *J–V* curves of the tensile-strained and the strain-free devices. To avoid possible misleading due to sample variation, we fabricated 40 cells under optimal conditions in each batch. Figure 3a shows the histograms of PCEs for each batch of samples with/without strain. The tensile-strained devices exhibited the PCE averaged around 18.7% with a wider distribution from 17.3% and 20.3%. In comparison, the strain-free devices achieved the averaged PCE of 19.8%, whose PCEs were distributed in a narrow range between 18.8% and 20.7%. The narrow distribution in PCEs of the strain-free devices stands for the good processing reproducibility. We also conducted the current–voltage (*I–V*) measurement for devices under different annealing conditions to preserve different tensile strains in the absorbers, which provided the statistics of parameters in Supplementary Fig. 11 and Supplementary Table 4. It is found that the fill factor (FF) and the open-circuit voltage (*V*_OC_) have significantly improved with the increase of the flipped annealing time, wherein the surface tensile strain is gradually released through prolonged annealing at 120 °C.

We measured current-density voltage (*J–V*) characteristics of one of the best devices under a simulated illumination of air mass (AM) 1.5 and 100 mW cm^−2^. This device generated a short-circuit current density (*J*_SC_) of 22.8 mA cm^−2^, a FF of 78.0%, an open-circuit voltage (*V*_OC_) of 1.17 V, and a power conversion efficiency of 20.7% (Fig. 3b). The forward and reverse scanning current density–voltage (*J–V*) curves showed negligible hysteresis in the corresponding device in Supplementary Fig. 11e, which was likely attributed to the improved carrier extraction at the interface^44,45^. By holding a bias near the maximum power output point (0.96 V), a stabilized photocurrent of 21.3 mA cm^−2^ was obtained, corresponding to a stabilized efficiency of 20.5% (the inset of Fig. 3b). The device performance was certified by the independent third party (Supporting Information). The External quantum efficiency (EQE) spectra for the two types of devices were shown in Supplementary Fig. 11f. Compared to the tensile strain device, the strain-free device showed improved light harvesting efficiency along the entire absorption wavelength range of 350 to 800 nm. The integrated photocurrent densities were calculated to be 20.81, 22.7 mA cm^−2^, respectively, which was in good agreement with the *J*_SC_ derived from the *J–V* measurement. Thus far, we observed the significant improvement in FF and *V*_OC_ in strain-engineered devices. This is likely attributed to improved carrier extraction dynamics around the absorber interface, which is subjected to further analysis.

Firstly, the carriers transport behavior at the interface was probed with transient photocurrent (TPC) and time-resolved photoluminescence (TRPL) measurement. The TPC measurement (Fig. 3c) was often used to monitor the carrier transport across the device. By fitting with the exponential function, the photocurrent decay time was significantly reduced from 12.96 to 1.0 µs (Supplementary Table 5). A faster decay of photocurrent than the reference device suggested the improvement in carrier extraction when tensile strain gradient was almost eliminated at the surface. Further investigation with the TRPL measurement indicates improved hole extraction due to elimination of residual strain, as observed in the samples with the configuration of glass/perovskite/Spiro-OMeTAD. As shown in Supplementary Fig. 12 and Supplementary Table 6, the average decay time *τ*_avg_ related to PL quenching was dramatically decreased from 14.6 to 4.9 ns in the strain-free sample, showing the higher quenching rate. It indicates that the strain-free sample exhibits better hole extraction at the interface between absorber and hole transport material (HTM), as compared to that of the tensile strain sample. This result is in accordance with the TPC measurement, showing that modulation of residual tensile strain can accelerate the carrier transfer process.

Secondly, we investigated how the carrier recombination process of devices is affected by the residual strain. We conducted the combined measurements of light-intensity-dependent *V*_OC_, electrochemical impedance spectroscopy (EIS) and transient photovoltage decay (TPV). The light-intensity-dependent *V*_OC_ provides critical insights into the mechanism of recombination processes in solar cells^46^. The corresponding charge carrier recombination process is reflected by the ideality factor of ‘‘*n*’’ as determined by the slope of the *V*_OC_ versus incident light-intensity according to the equation *V*_OC _= *E*_g_/*qnk*_B_*T*/*q*ln*J*_0_/*J*; where *q* is the elementary charge, *k*_B_ is the Boltzmann constant, and *T* is the temperature. When the ideality factor *n* approaches 2, Shockley-Read-Hall (SRH) type, trap-assisted recombination dominates. As shown in Fig. 3d, from the relationship between *V*_OC_ ~ ln(I), the ideality factor *n* are 1.01, 1.55 for the devices with/without tensile strain, respectively. It indicates that trap-assisted SRH recombination is effectively suppressed by reducing the tensile strain that is mainly located at the absorber surface. The alleviated SRH recombination may be attributed to the reduced trap density in strain-free devices, wherein crystal structure homogeneity is achieved.

The carrier dynamics across the perovskite/HTM interface upon strain modulation is further examined by EIS. Corresponding Nyquist plots were obtained from solar cells with/without gradient residual strain in dark without applied bias (Fig. 3e). It shows two separate arcs, equivalent to the resistive and capacitive components of various interfaces^47,48^. Generally, the first arc in the intermediate-frequency region is associated with the recombination impedance due to the selective contacts or their interface with the perovskite active layer in the device. Here, we mainly focused on the mid-frequency region and found that the recombination impedance increased from 23.69 to 521.10 KΩ when the tensile strain was relaxed. It is consistent with that of the light-intensity-dependent *V*_OC_ measurement. We further measured the recombination resistance (*R*_rec_), wherein the device was subjected to applied bias ranging from 0.4 to 1.0 V. By fitting with the simple RC equivalent circuit, we exacted the recombination resistance under different bias as shown in Fig. 3f. It is clear that strain-free devices display a larger *R*_rec_ than that of the tensile-strained one, which indicates that the interfacial charge recombination is suppressed in strain-free device. The reduced recombination loss was further confirmed by TPV measurement^49^, wherein the photovoltage decay time was significantly increased from 4.01 to 10.32 µs (Supplementary Fig. 12 and Supplementary Table 5) when tensile strain was released. It is also in agreement with higher *V*_OC_ observed in the devices with strain-free absorbers.

### Mechanisms of the effect of strain on photovoltaic properties

**Fig. 4:**
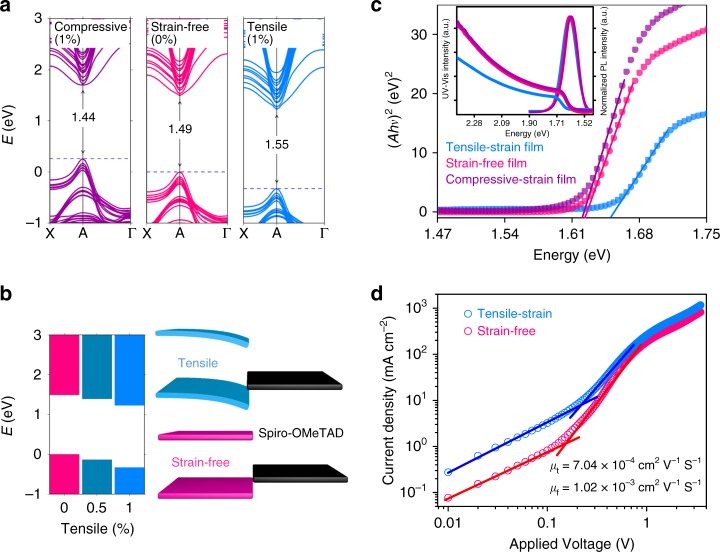
Strain-induced electronic structure analysis. **a** Calculated band structures under biaxial tensile, zero, and compressive strains from first-principle density functional theory (DFT)-based approaches. The band structure alignment is made by using the vacuum energy level as reference. **b** The evolution of band-edge energies under gradually increasing tensile strains in perovskite films (left panel), and the schematic of the band alignment between tensile strain/strain-free film and hole transport layer in solar cell. **c** Ultraviolet (UV)–visible (Vis) absorption spectra and PL spectra under tensile strain, strain-free, and compressive strain conditions. **d** The *J–V* characteristics of the hole-only space-charge-limited current (SCLC) device with/without residual tensile strain

The above results unambiguously indicate that the gradient distribution of residual strains directly affects the hole carrier dynamics across the perovskite/HTM interface and thus the device performance. To reveal the underlying mechanism, we performed first-principles calculations to simulate changes of electronic structure and optoelectronic properties of the perovskite films under strained condition. In particular, we chose the (001)-oriented FAPbI_3_ perovskite film represented by six-layers Ruddlesden-Popper phase of FAPbI_3_ and applied in-plane biaxial strains to it. Since (001) is one of predominate growth direction of perovskite crystals as indicated by the XRD measurements, and FAPbI_3_, MAPbBr_3_ and their mixture have similar electronic structures^50,51^, we expect the above model can reasonably mimic the strained mixed perovskite (FAPbI_3_)_0.85_(MAPbBr_3_)_0.15_ films in experiment. We embedded the (001) FAPbI_3_ film in the vacuum and applied biaxial strains of 1%, 0.5% (compressive) and –0.5%, –1% (tensile), respectively (Supplementary Fig. 13). We found that the perpendicular direction exhibit quite small opposite stain components as the rotation of organic FA/MA molecules contributes substantially to strain relaxation. The calculated band structures under tensile, zero, and compressive strains are shown in Fig. 4a, b. One sees that the band gaps of the films show increase with the strain changing from compression, zero-strain, to tension. This agrees with the observed tendency of bandgap change from the ultraviolet (UV)–visible (Vis) absorption and PL measurement (Fig. 4c). To be noted, the bandgap measured from UV–Vis absorption and PL spectra less than the theoretical value. It is because the theoretical value was calculated with the assumption that the entire lattice structure is subjected to strain state. In our experiment however, the largest strain only locates at the surface region rather than the entire film (Fig. 1c), wherein quite a large portion of films remain the original bandgap. The tendency can be explained by consideration of the band-edge features of Pb halide perovskites APbX_3_, where the valence band (VB) edge is composed of the strong anti-bonding interaction between Pb-*s* and X-*p* orbitals, and the conduction band (CB) edge is predominantly from Pb-*p* orbital with weak anti-bonding character^51,52^. With the perovskite film experiencing compressive, zero, to tensile biaxial strain, the in-plane lattice gradually expands, which weakens the Pb–X bonds and thus in principle pulls down both the anti-bonding VB and CB energy levels. However, the VB with strong anti-bonding hybridization is substantially decreased, while the CB is less affected. As the result, the bandgap shows increase from compressive, zero, to tensile strain. This explanation is indeed supported by our calculations as shown in Fig. 4a.

Based on the changed electronic structure in the strained perovskite, we attempt to illustrate how residual strains affect hole carrier dynamics in solar cells. Figure 4b (left panel) shows the evolution of band-edge energies under tensile strains. The CB decreases slightly with increasing strain magnitude, whereas the VB exhibits pronounced downshift. It reveals that strains evolve vertically in the perovskite film, wherein the largest tensile strain is observed at the perovskite/HTM interface. Therefore, the VB bends downward monotonously over the entire perovskite absorber layer, as depicted in the Fig. 4b (right panel). This VB downward bending has dual effects on the hole carrier dynamics. On one hand, it creates the ‘‘cliff-type’’ band alignment by repelling hole carrier energy level away from that of the HTM (Fig. 4b). This was reported to be unfavorable for hole extraction in Cu(In,Ga)Se_2_^53^ and perovskite solar cells^54–58^. On the other hand, the hole mobility is possibly affected along the direction of VB downward bending, wherein the hole carrier diffusion suffers from extra hindering force field due to unfavored energy level gradient. As indicated by the space-charge-limited current (SCLC) measurement (Fig. 4d) of the fabricated capacitor-like devices by sandwiching the perovskite films between ITO and Au, the mobilities of the samples with and without tensile strain were calculated to be 7.04 × 10^−4^  and 1.02 × 10^−3^ cm^2^ V^−1^ S^−1^, respectively. Indeed, the experimentally observed carrier mobility is improved when tensile strain is released. In short, by eliminating the tensile strain gradient in the perovskite film, the VB is flattened to cancel the ‘‘cliff-type’’ band alignment of perovskite absorber/HTM and hole mobility is enhanced simultaneously. It thus favors the charge transport and extraction of photogenerated holes, which suppresses the carrier recombination and leads to the significant improvement in FF and *V*_OC_ in the corresponding device.

In addition to the above two main effects, the tensile strain induced downward shift of valence bands may also result in the deeper defect levels of the perovskite films with the assumption of defect energy levels being not sensitive to strain. This is supported by the experimental observation that the hydrostatic pressure render the shallower defect energy levels of hybrid halide perovskites^59,60^. As demonstrated above we also observed the prolonged carrier lifetime in the strain-free samples (Supplementary Fig. 14).

## Discussion

In conclusion, we revealed gradient evolution of residual strain in the vertical direction of the mixed halide perovskite film by depth-dependent grazing incident X-ray diffraction characterization. The residual strain distribution may be stemmed from composition inhomogeneity and/or gradient thermal stress during film processing. A simple technique was developed to modulate the strain nature (e.g., tensile and compressive) and its gradient over the perovskite film in a controllable manner. This allows us to identify substantial impact of the residual strain gradient on hole carrier dynamics across the perovskite solar cell. First-principle calculations reveal that the strain gradient induces valence bands bending of perovskite absorber and thus affects the interfacial hole dynamics. By reducing the strain gradient of the perovskite film through strain engineering, we achieved substantial improvement in hole carrier transport and extraction across the interface of perovskite absorber/HTM. Consequently, the optimized perovskite solar cell reaches the certified power conversion efficiency of 20.7%. By demonstrating the impact of residual strain on optoelectronic properties of halide perovskite film, this contribution sheds a light on further understanding the composition-structure-property relationship of halide perovskite system, which may be exploited to further advance the performance of halide perovskite based optoelectronic devices.

## Methods

### Materials

All the commercial materials were used as received without further purification, including ethanol (AR Beijing Chemical Works), Methylamine (33 wt.% in absolute ethanol), Formamidine acetate (99%, Aldrich), HBr (48 wt.% in water, Sigma-Aldrich), HI (57 wt.% in water, Sigma-Aldrich), PbI_2_ (99.999%, Sigma-Aldrich), PbBr_2_ (99.999%, Aldrich), CsI (99.90%, Aladdin Industrial Corporation) N,N-dimethylformamide (DMF, 99.99%, Sigma-Aldrich), Dimethyl sulfoxide (DMSO, 99.9%, Sigma-Aldrich), chlorobenzene (CB, 99.9%, Sigma-Aldrich), Spiro-OMeTAD (Lumtec), bis(trifluoromethane)sulfonimide lithium salt (99.95%, Aldrich), 4-tert-butylpyridine (99.9%, Sigma-Aldrich), acetonitrile (99.9%, Sigma-Aldrich), and ITO substrates. The HC(NH_2_)_2_I and CH_3_NH_3_Br were prepared according to procedure mentioned in previous work.

### Perovskite precursor solutions

The precursor solutions were prepared according to the work delivered by Saliba.

FAMA perovskite solution: The mixed perovskite solution were prepared by mixing FAI (1.38 M), PbI_2_ (1.49 M), MABr (0.24 M), PbBr_2_ (0.26 M) in anhydrous DMF: DMSO 4:1 (v:v) according to previous work with a slight amount of excessive PbI_2_. To be convenient, we labeled the mixed perovskite solution with compositions mentioned above as FAMA^57^.

CsI solutions: CsI solution was deposited by dissolving CsI in pure DMSO with the concentration of 1.5 M.

(FAMA)_(100-*x*)_Cs_*x*_ perovskite solution: (FAMA)_(100-*x*)_Cs_*x*_ perovskite solution was obtained by adding appropriate amount of CsI into 300 μL FAMA perovskite solution with different cesium concentrations (volume ratio, *x* = 100% × *V*_CsI_/(*V*_CsI_ + 300)) to achieve the desired cation composition 5%.

### Optimal flipped annealing method for polycrystalline perovskite film

The perovskite film washed by antisolvent was annealed about 20 min at 120 °C through the normal method in order to most of the solvent can escape smoothly, then it was flipped over and annealed 25 min at 120 °C so that the maximum tensile strain in the top surface can be gradually released at the highest temperature. In order to drop down the cooling rate at the same total annealing time, we first transferred the film to a hot stage at 80 °C for 1 min and then transferred to a hot stage at 40 °C for 1 min. All annealing processes are finished in in a nitrogen glove box.

### Sample preparation and devices fabrication

The ITO substrate was sequentially washed with distilled water, acetone, ethanol, and isopropanol. After 30 min of UV–O_3_ treatments, the SnO_2_ electron transport layers (ETLs) were spin-coated on ITO substrates from the SnO_2_ colloidal solutions, and annealed on a hot plate at the displayed temperature of 150 °C for 30 min in ambient air. For the mixed A-cation FA_0.85_MA_0.15_Pb(I_0.85_Br_0.15_)_3_ and FA_0.85_MA_0.15_Cs_0.05_Pb(I_0.85_Br_0.15_)_3_ metal halide perovskite layer, one-step method used toluene as antisolvent developed by Saliba was adopted. In detail, the perovskite solutions were spin-coated at 6000 rpm for 30 s. Two-hundred microliters of chlorobenzene was dropped on the spinning substrate during the spin-coating step at 10 s before the end of the procedure. Then, the as-fabricated film were baked at 120^ ο^C for 45 min in a nitrogen filled glove box. After the perovskite annealing, 30 μL Spiro-OMeTAD solution doped with LiTFSI and tBP was deposited at 3000 rpm for 30 s. The hole transport material (HTM) solution was prepared by dissolving 60 mg spiro-OMeTAD, 30 μL 4-tert-butylpyridine and 35 μL Li-TFSI/acetonitrile (260 mg mL^−1^) in 1 mL chlorobenzene. Finally, 100 nm Ag was thermally evaporated as counter electrode under a pressure of 5 × 10^−5^ Pa on top of the hole transport layer to form the back contact.

### Fabrication of space-charge limited current devices

The structure of the device was ITO/PEDOT:PSS/perovskite/Spiro-OMeTAD /Au. The ITO glass substrate was treated by UV–O_3_ for 30 min, the PEDOT:PSS was filtered and then spin-coated on ITO substrates. FA_0.85_MA_0.15_PbI_2.55_Br_0.45_ perovskite film was deposited on the PEDOT:PSS/ITO substrate by spin-coating. After the perovskite annealing, Spiro-OMeTAD layer was deposited and 150 nm Au was thermally evaporated as counter electrode.

### Characterizations

The morphologies and sizes of nanocrystals were characterized by JEOL JEM-2100 transmission electron microscopy (TEM). Scanning electron microscope (SEM) images were measured using Hitachi S4800 field-emission scanning electron microscopy. XRD patterns were recorded on a Rigaku smartlab X-ray Diffractometer. The cross-section of the device was prepared by focused ion beam (FIB) using a FEI Helios Dualbeam system. The sample was first covered by Pt protection layer deposited by electron beam and ion beam in dualbeam system, and was then milled to thin lamella following standard FIB sample preparation techniques. Due the beam-sensitivity of organic–inorganic hybrid halide perovskite structure, low beam voltage, and current was applied during final cleaning steps. The as-prepared thin lamella was studied by high angle annular dark field scanning transmission electron microscopy (HAADF-STEM) using a FEI Titan G2 microscope equipped by an aberration corrector for probe forming lens, operated at 300 kV. Depth-dependent steady-state photoluminescence (PL) measurement was executed by confocal fluorescence microscope and time-resolved photoluminescence data was obtained by FLS980 (Edinburgh Instruments Ltd) with an excitation at 465 nm. The time-of-flight secondary ion mass spectrometry (TOF-SIMS) measurements (Model TOF-SIMS V, ION-TOF GmbH) were performed with the pulsed primary ions from a Cs+ (3 keV) liquid-metal ion gun and a Bi+ pulsed primary ion beam for the analysis (25 keV). Current–voltage characteristics were recorded by using a Keithley 2400 source-measure unit. The typical current–voltage characteristics of the devices were measured using a Keithley 2400 source meter by reverse scanning from 1.2 to −0.2 V or forward scanning from −0.2 to 1.2 V at a scanning speed of 50 mV s^−1^. The photocurrent was measured under AM1.5G illumination at 100 mW cm^−2^ under a by 3A steady-state solar simulator (SS-F5-3A). Light-intensity was calibrated with a National Institute of Metrology (China) calibrated KG5-filtered Si reference cell. The effective area of each cell was 0.102 cm^2^ defined by masks for all the photovoltaic devices discussed in this work. Space-charge limited current measurements were conducted on hole-only ITO/PEDOT:PSS/perovskite/Spiro-OMeTAD/Ag devices. A Keithly 2400 source meter was used to measure the relevant *J–V* curves. The absorption spectra were measured by Hitachi UH4150 spectrophotometer. External quantum efficiencies (EQE) were performed on a solar cell quantum efficiency measurement system (QE-R) supported by Enli Technology Co., Ltd. A calibrated silicon diode with a known spectral response was used as a reference. The transient photovoltage/photocurrent (TPV/TPC) decay measurements were obtained on Molex 180081-4320 simulating one sun working condition, the carriers were excited by a 532 nm pulse laser. The electrochemical impedance spectroscopy (EIS) was determined by the electrochemical workstation (Germany, Zahner Company), employing light emitting diodes driven by Export (Germany, Zahner Company).

### First-principles calculations

Calculations were performed within the framework of density functional theory (DFT) by using plane-wave pseudopotential methods as implemented in the Vienna Ab initio Simulation Package^61,62^. The electron−ion interactions were described by the projected augmented wave pseudopotentials^63^ with the 1*s* (H), 2*s* and 2*p* (C), 2*s* and 2*p* (N), 5*s* and 5*p* (I) and 5*d*, 6*s* and 6*p* (Pb) electrons treated explicitly as valence electrons. We used the generalized gradient approximation formulated by Perdew, Burke, and Ernzerhof^64^ as the exchange correlation functional. Kinetic Energy cutoff for the plane-wave basis set were set to 400 eV. The k-point meshes with grid spacing of 2*π* × 0.03 Å^−1^ were used for electronic Brillouin zone integration. We modeled the perovskite film by using the (001)-oriented six-layers Ruddlesden-Popper phase of FAPbI_3_ embedded in a vacuum region of 30 Å. The biaxial strains of 1%, 0.5% (compressive) and –0.5%, –1% (tensile) were applied to the film, respectively. The structures under the biaxial strain conditions were optimized by fixing the in-plane structure parameters of Pb–I perovskite framework through total energy minimization with the residual forces on the atoms converged to below 0.02 eV Å^−1^. To properly take into account the long-range van der Waal interactions that play a nonignorable role in the hybrid perovskites involving organic molecules, the vdW-optB86b functional^65^ was adopted. The alignment of the band structures upon different strains is made by using the vacuum energy as reference.

## Supplementary information

Supplementary Information

## Data Availability

All the relevant data are available from the corresponding authors upon reasonable request.
