# Supplementary material for: Strain engineering in perovskite solar cells and its impacts on carrier dynamics
Source: Nat Commun. 2019 Feb 18;10:815. doi: 10.1038/s41467-019-08507-4 (PMC6379394; doi:10.1038/s41467-019-08507-4)
Supplement: Supplementary file 1 — Supplementary Information [file 41467_2019_8507_MOESM1_ESM.docx]

**Supplementary Information**

Cheng Zhu^1^**^🟉^**, Xiuxiu Niu^1^**^🟉^** , Yuhao Fu^3^**^🟉^**, Nengxu Li^2^, Chen Hu^8^, Yihua Chen^2^, Xin He^3^, Guangren Na^3^, Pengfei Liu^1^, Huachao Zai^5^, Yang Ge^4^, Yue Lu^4^, , Xiaoxing Ke^4^, Yang Bai^1^, Shihe Yang^7,8^, Pengwan Chen^6^, Yujing Li^1^, Manling Sui^4^, Lijun Zhang*^3^, Huanping Zhou*^2^, Qi Chen*^1^

^1^Beijing Key Laboratory of Nanophotonics and Ultrafine Optoelectronic Systems, School of Materials Science & Engineering, Beijing Institute of Technology, Beijing 100081, China

^2^Department of Materials Science and Engineering, College of Engineering, Peking University, Beijing 100871, China

^3^State Key Laboratory of Superhard Materials, Key Laboratory of Automobile Materials of MOE, and School of Materials Science and Engineering, Jilin University, Changchun 130012, China

^4^Institute of Microstructure and Properties of Advanced Materials, Beijing University of Technology, Beijing 100124, China

^5^Department of Materials Science and Engineering, College of Science, China University of Petroleum, Beijing 102249, China

^6^State Key Laboratory of Explosion Science and Technology, Beijing Institute of Technology, Beijing 100081, China

^7^Guangdong Key Lab of Nano-Micro Material Research, School of Chemical Biology and Biotechnology, Shenzhen Graduate School, Peking University, Xili University Town, Shenzhen 518055, Guangdong, China.

^8^Department of Chemistry, The Hong Kong University of Science and Technology, Clear Water Bay, Kowloon, Hong Kong, China

Correspondence and requests for materials should be addressed to or to L.Z. (email: [lijun_zhang@jlu.edu.cn](mailto:lijun_zhang@jlu.edu.cn)) or to H.Z. (email: [happy_zhou@pku.edu.cn](mailto:happy_zhou@pku.edu.cn)) or to Q.C. (email: [qic@bit.edu.cn](mailto:qic@bit.edu.cn)).


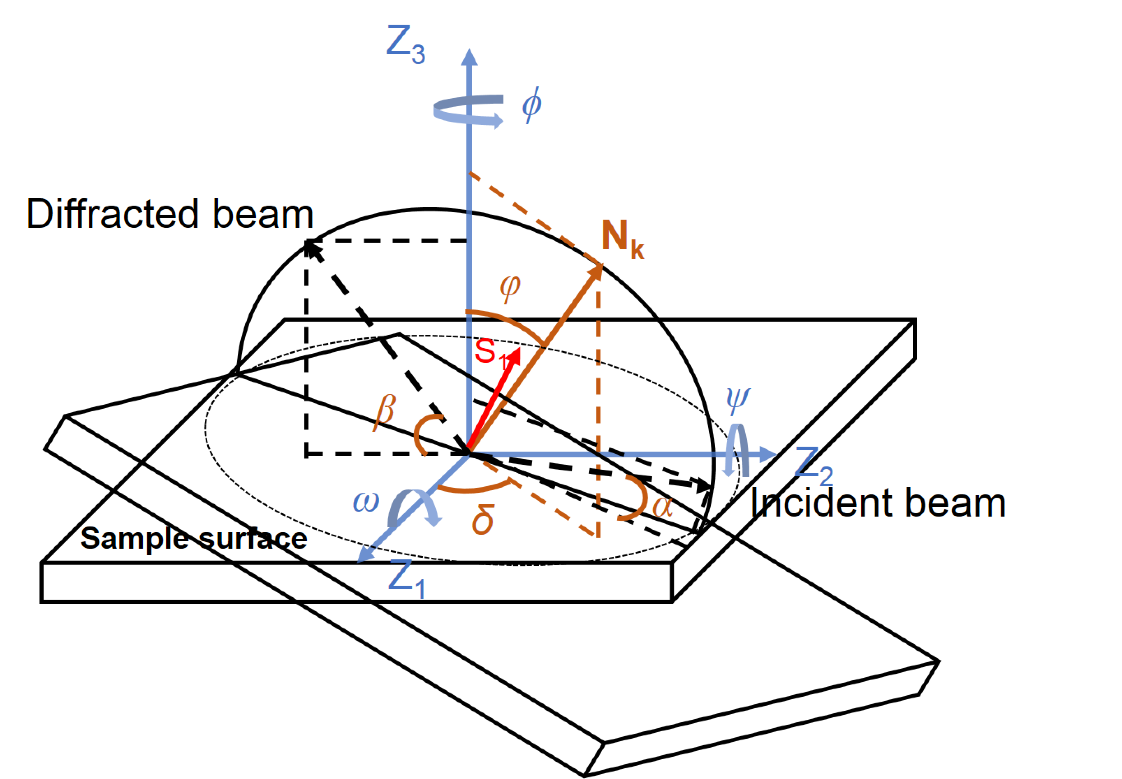


**Supplementary Figure 1** **|** Diffraction geometries of the depth-dependent strain distribution measurement. The relation between instrument reference (Z) frame and diffraction vector (*α* and *β* are the incident and exit angles of X-rays, defined as the angle between the incident/diffracted X-rays and the sample surface).


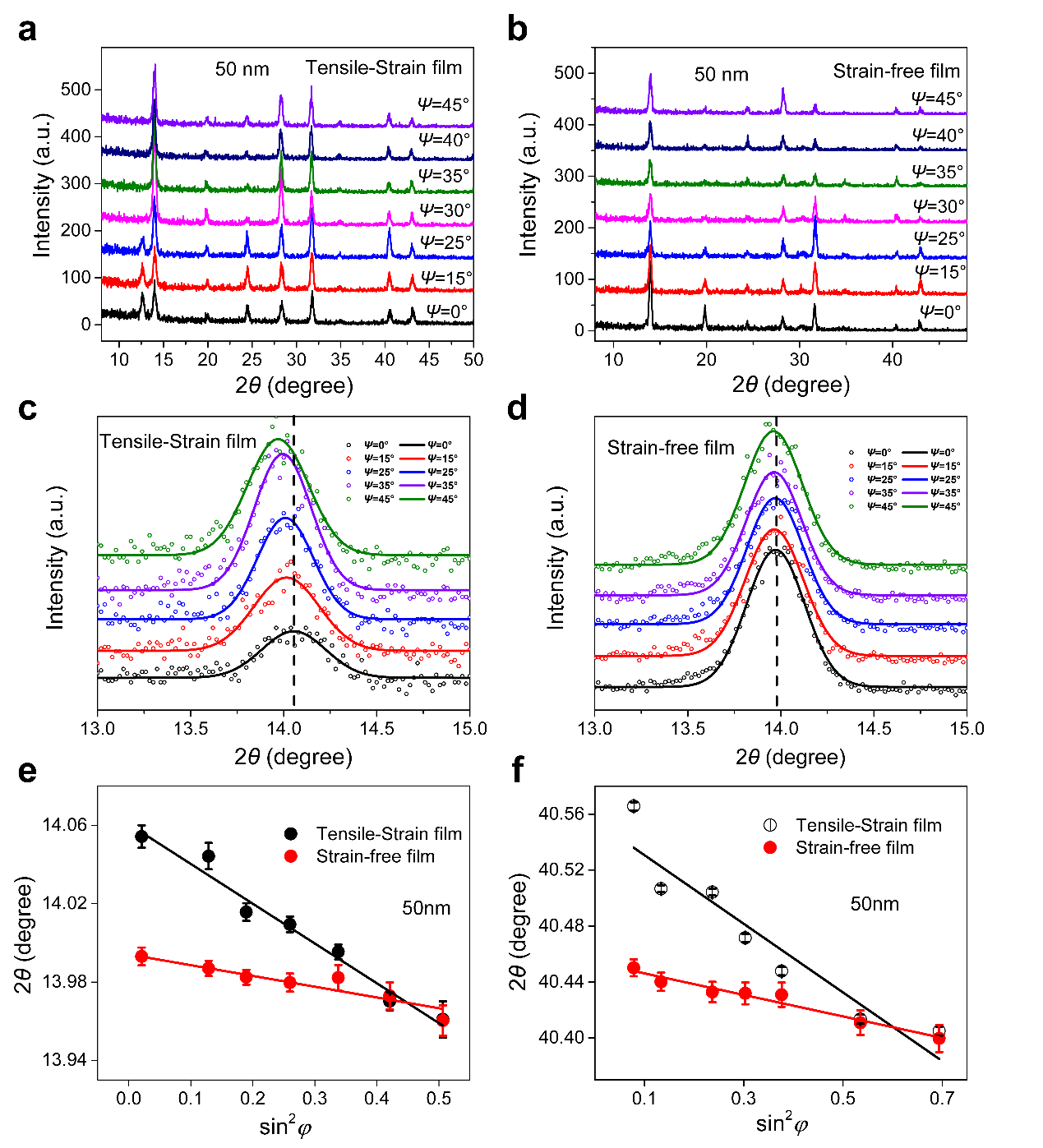


**Supplementary Figure 2** **| Results of residual strain measurement with the GIXRD method (a) (b)** GIXRD spectrum at different tilt angles at the depth of 50 nm for the tensile strained film, strain-free film **(c) (d)**XRD pattern of (001) crystal plane in 50 nm depth for the tensile strained film, strain-free film **(e) (f)** Residual strain distribution in the depth of 50 nm related to (001) (022) plane for the tensile strained film, strain-free film (measured (points) and Gauss fitted (line) diffraction strain data as a function of sin^2^*φ*).


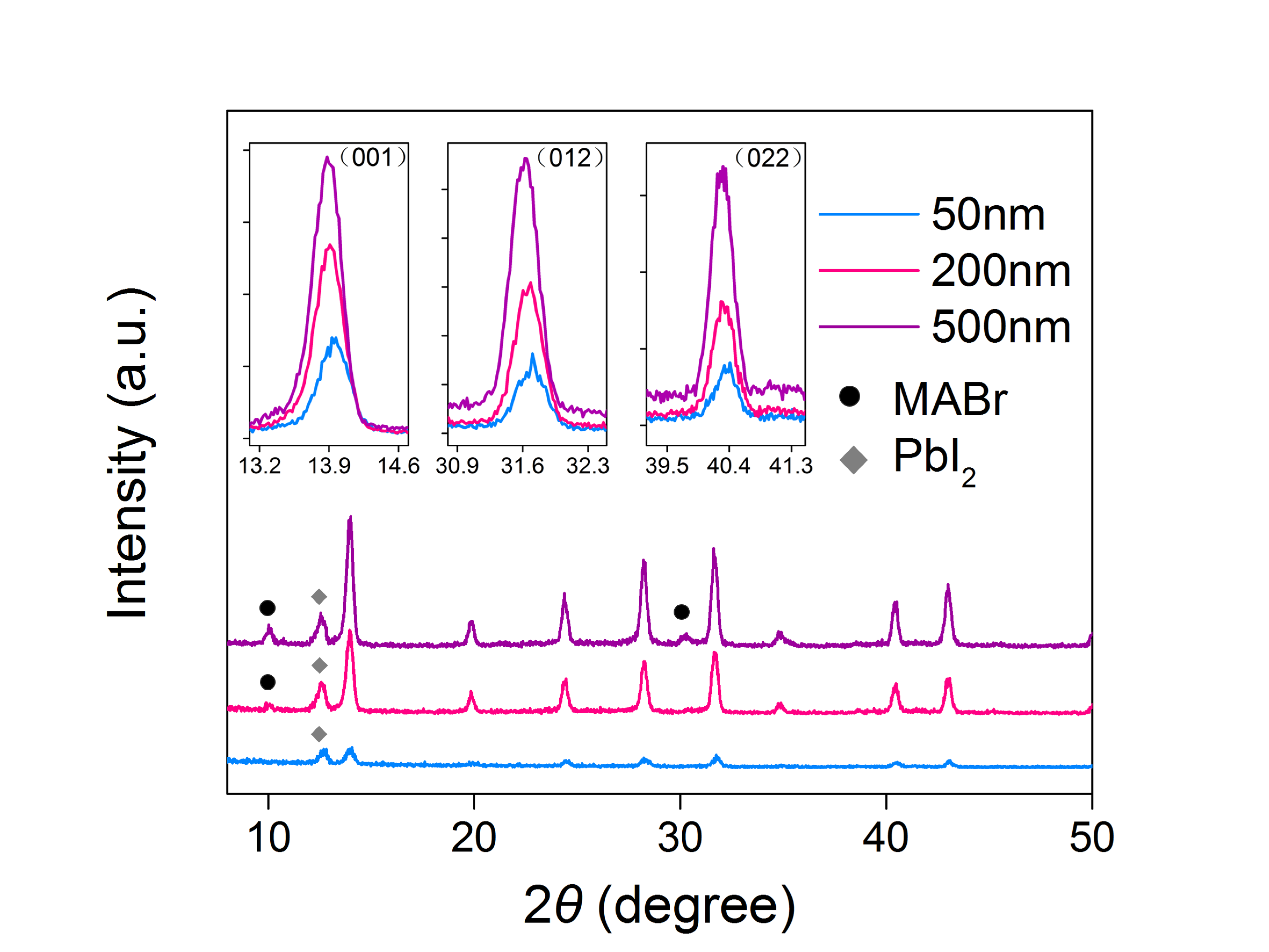


**Supplementary Figure 3** **|** X-ray diffraction spectrum at the depth of 50 nm, 200 nm, 500 nm in the perovskite film, the inset is the XRD pattern of (001) (012) (022) crystal plane in corresponding depth


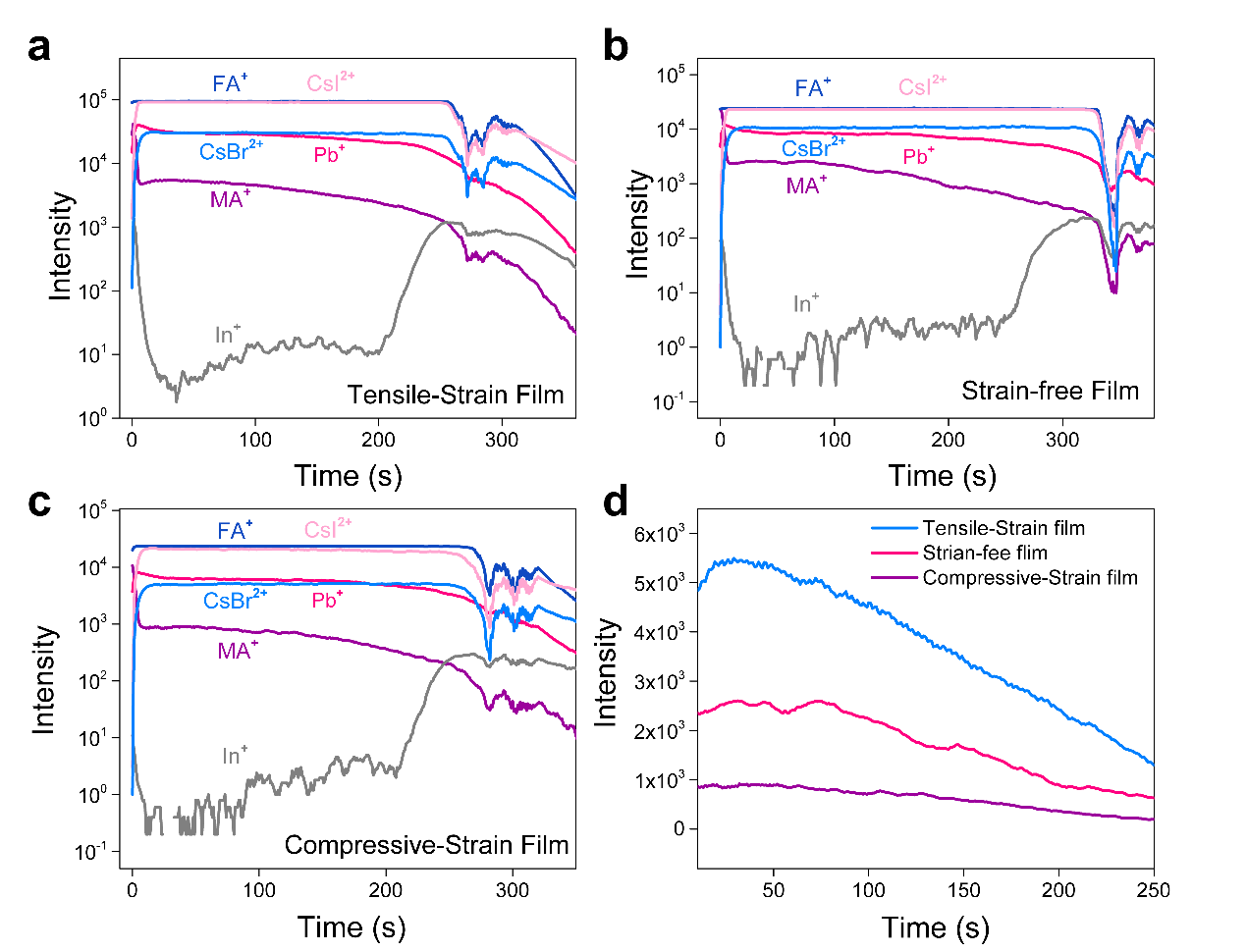


**Supplementary Figure 4** **|** **(a) (b) (c)** ToF-SIMS depth profiles of the (FAPbI_3_)_0.85_(MAPbBr_3_)_0.15_ perovskite film with tensile-strain, strain-free and compressive-strain. **(d)** ToF-SIMS depth profiles of the MA^+^ distribution with tensile-strain, strain-free and compressive-strain.


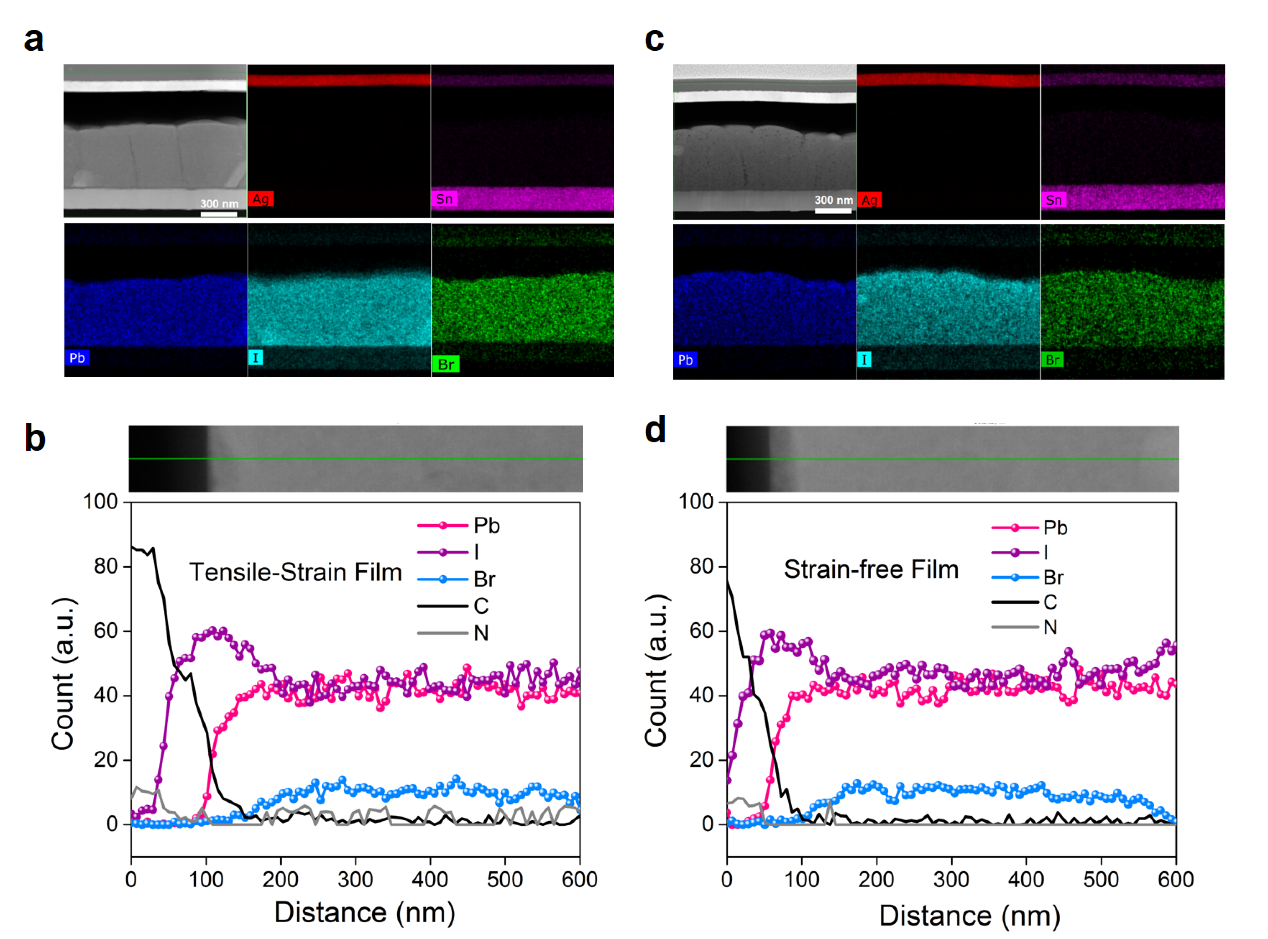


**Supplementary Figure 5 |** **(a) (b)** HAADF-STEM images, EDX maps and EDX line scan for element distribution of the tensile strained film **(c) (d)** HAADF-STEM images, EDX maps and EDX line scan for element distribution of the strain-free film.


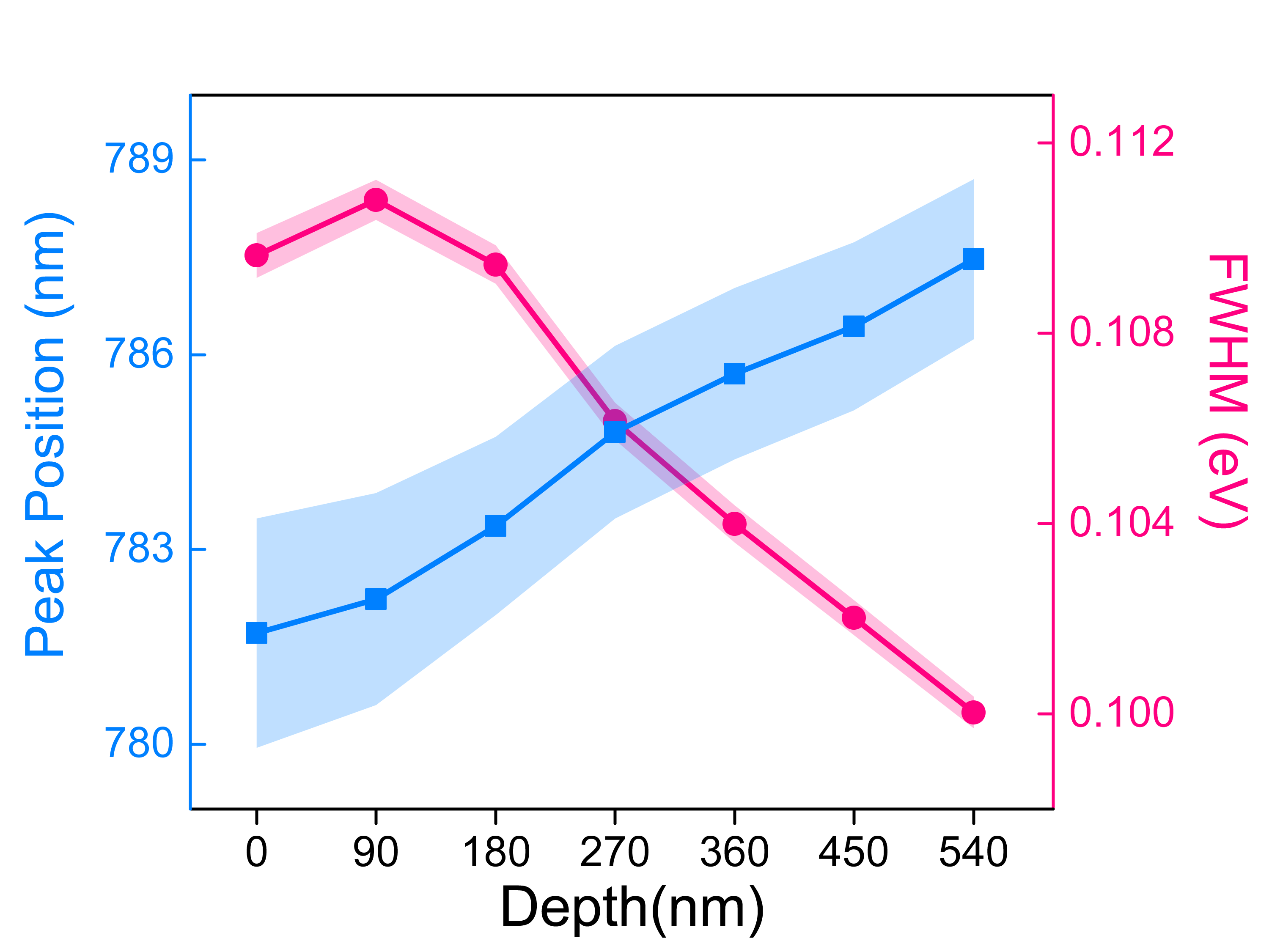


**Supplementary Figure 6** **|** PL peak position and FWHM as a function of film thickness for mixed perovskite according to PL depth profile of Confocal fluorescence microscope. The fill area indicates the standard deviation of the peak position and FWHM.


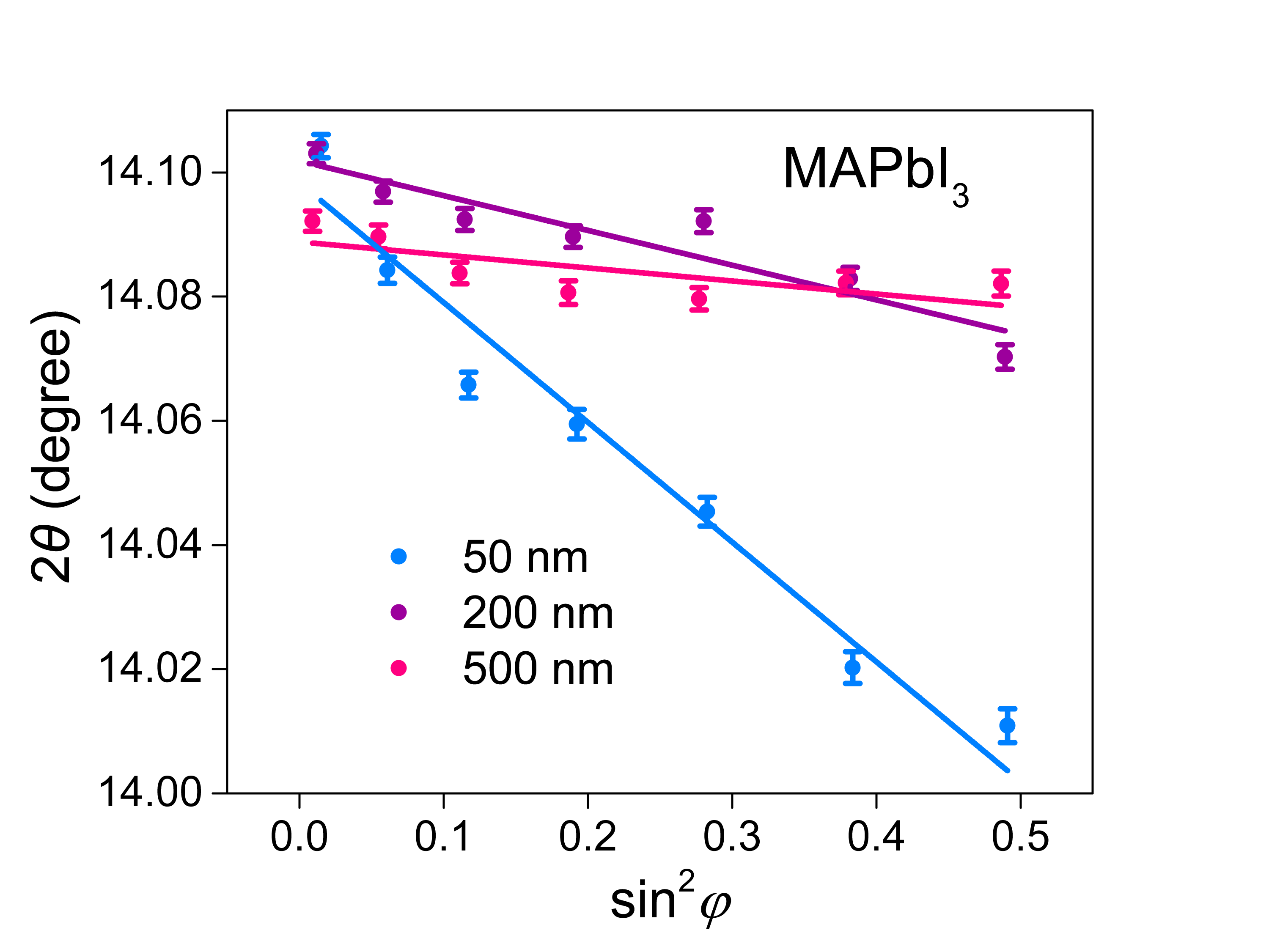


**Supplementary Figure 7** **|** Residual strain distribution in the different depth related to (011) plane for the tensile strained film (measured (points) and Gauss fitted (line) diffraction strain data as a function of sin^2^*φ*).


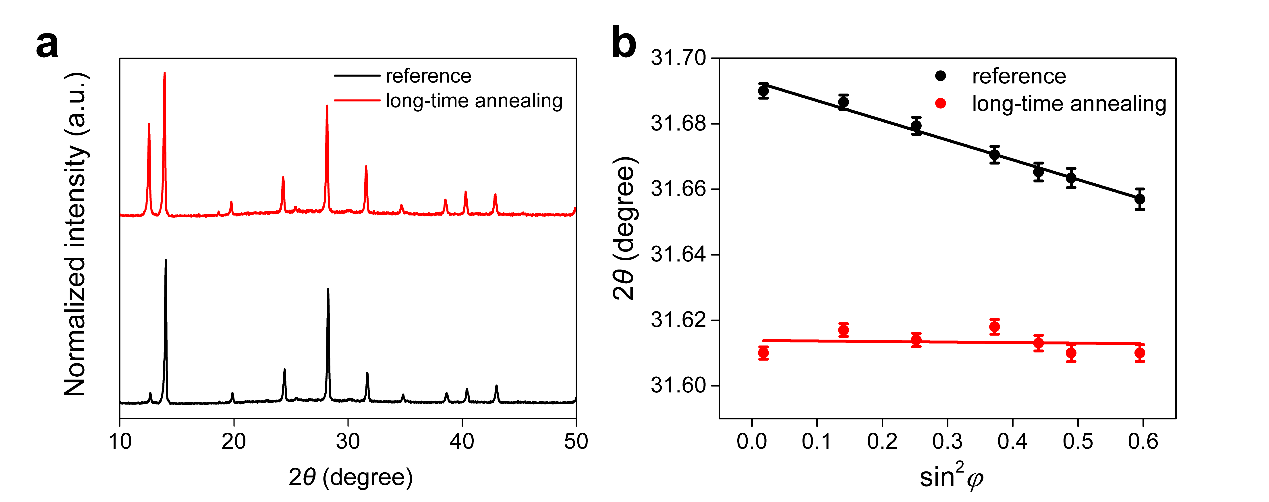


**Supplementary Figure 8** **| (a)** X-ray diffraction spectrum of the reference and the long-time annealing films **(b)** Residual surface strain distribution in the reference and the long-time annealing films (measured (points) and Gauss fitted (line) diffraction strain data as a function of sin^2^*φ*).


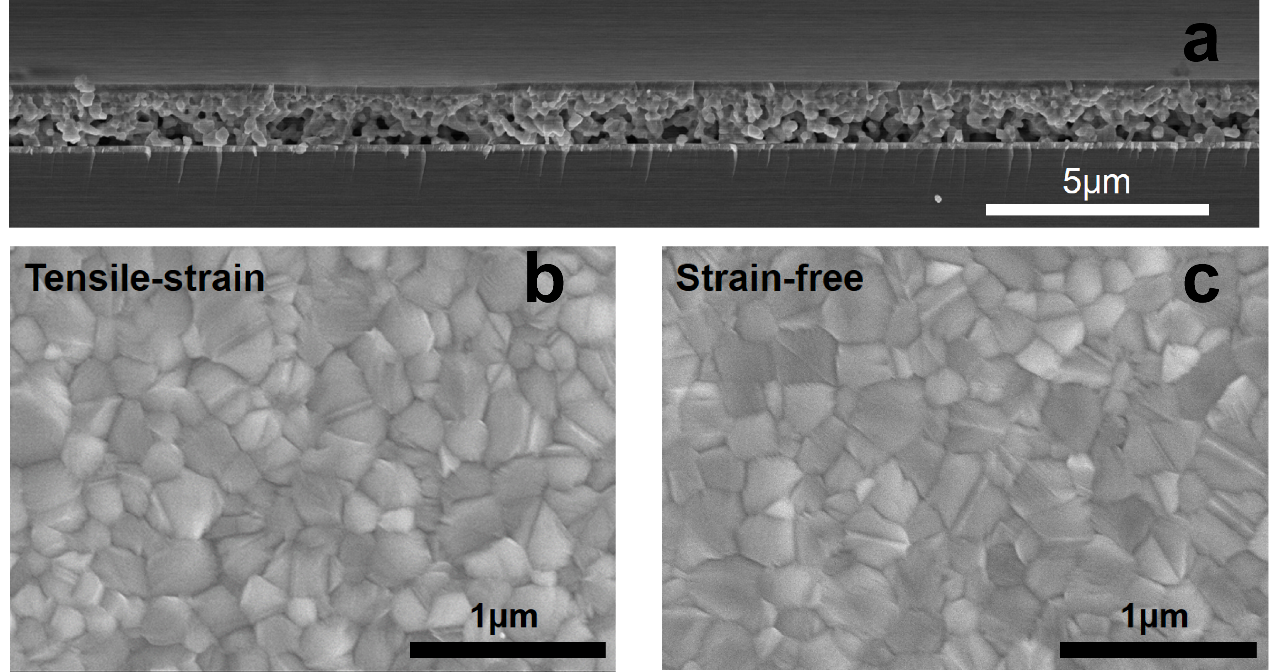


**Supplementary Figure 9 | (a)** The cross-sectional SEM image of compressive-strain film. A large number of holes were formed between perovskite layer and SnO2 layer due to the solvent cannot be spread smoothly. **(b) (c)** Results of SEM of the tensile strained and strain-free film.


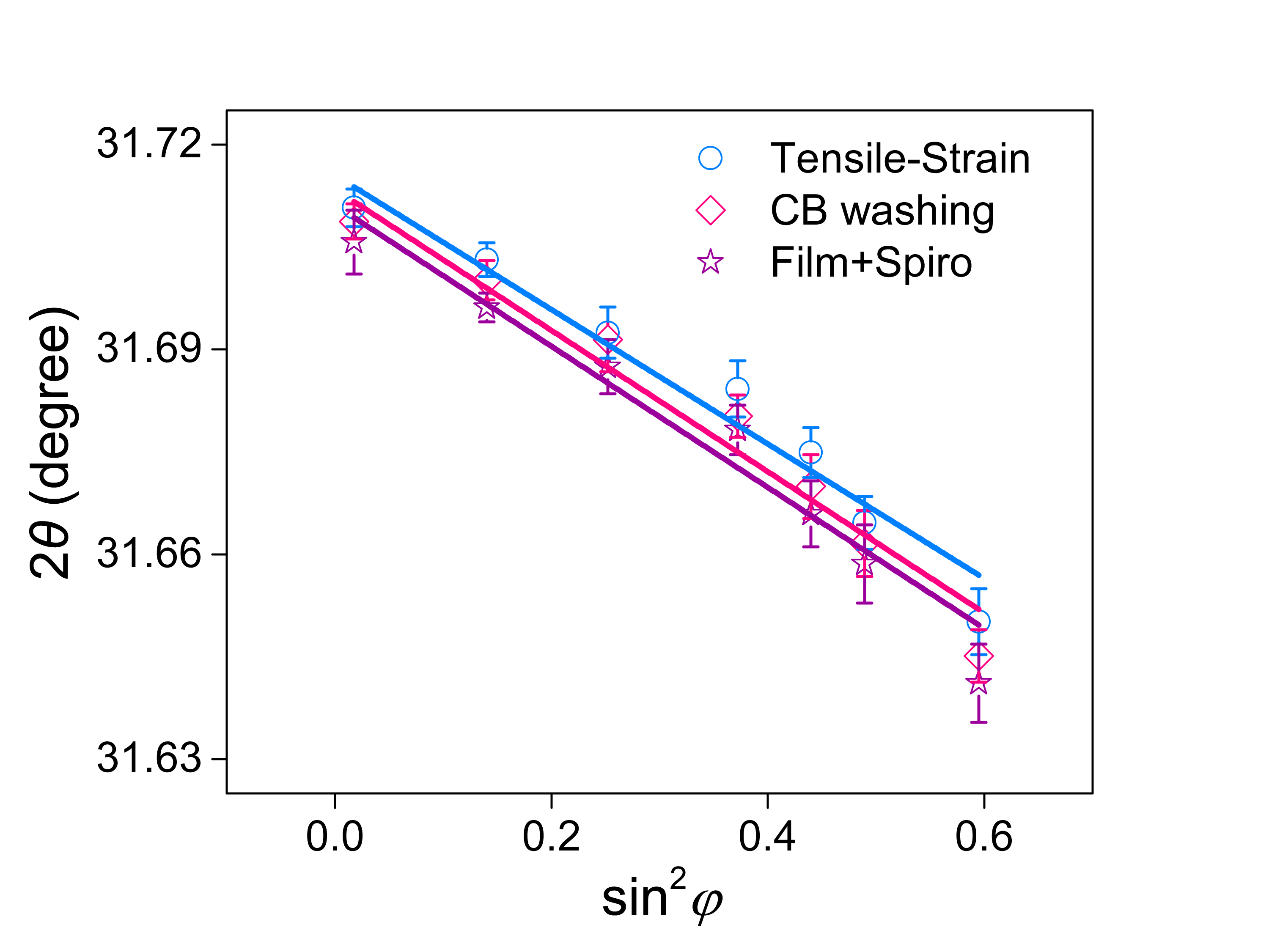


**Supplementary Figure 10** **|** Results of surface residual strain distribution with the GIXRD method at 50nm depth for reference sample, CB-dripped film and the film coated on Spiro-OMeTAD layer.


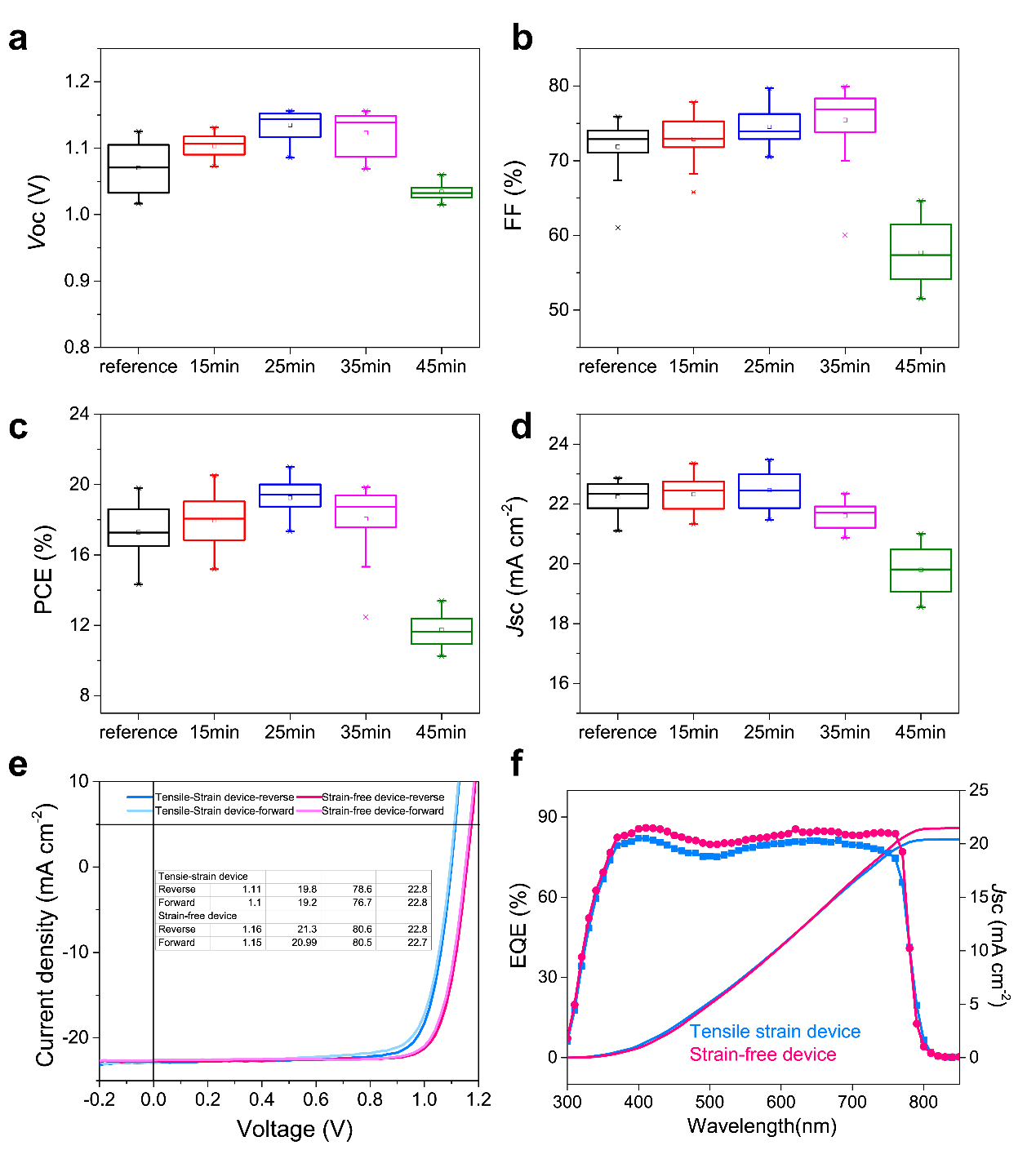


**Supplementary Figure 11 |** **(a) (b) (c) (d)** Statistics of *I-V* performance parameters (*V*oc, FF, PCE, *J*sc) for devices based on mixed FAMA perovskites with different flipped annealing time **(e)** *J-V* curve for the tensile-strain device and strain-free device under reverse and forward scan direction **(f)** External quantum efficiency (EQE) and integrated short-circuit current density for the tensile-strain device and strain-free device.


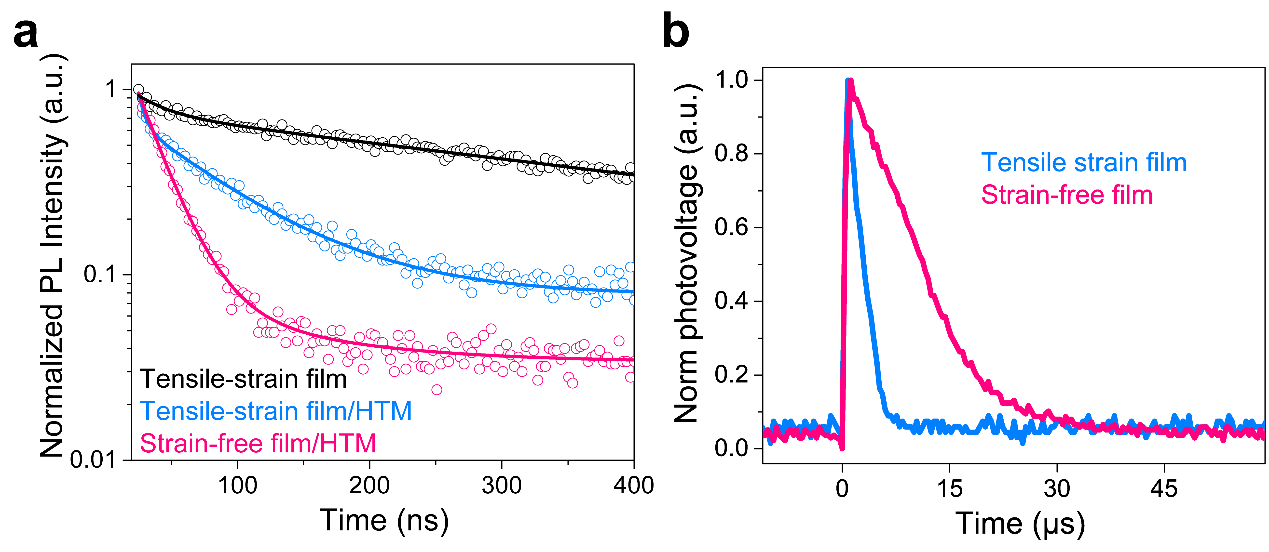


**Supplementary Figure 12 |** Charge extraction, recombination and transportation analysis for tensile strained and strain-free samples. **(a)** TRPL spectrum for films and **(b)** TPV decay line for devices.


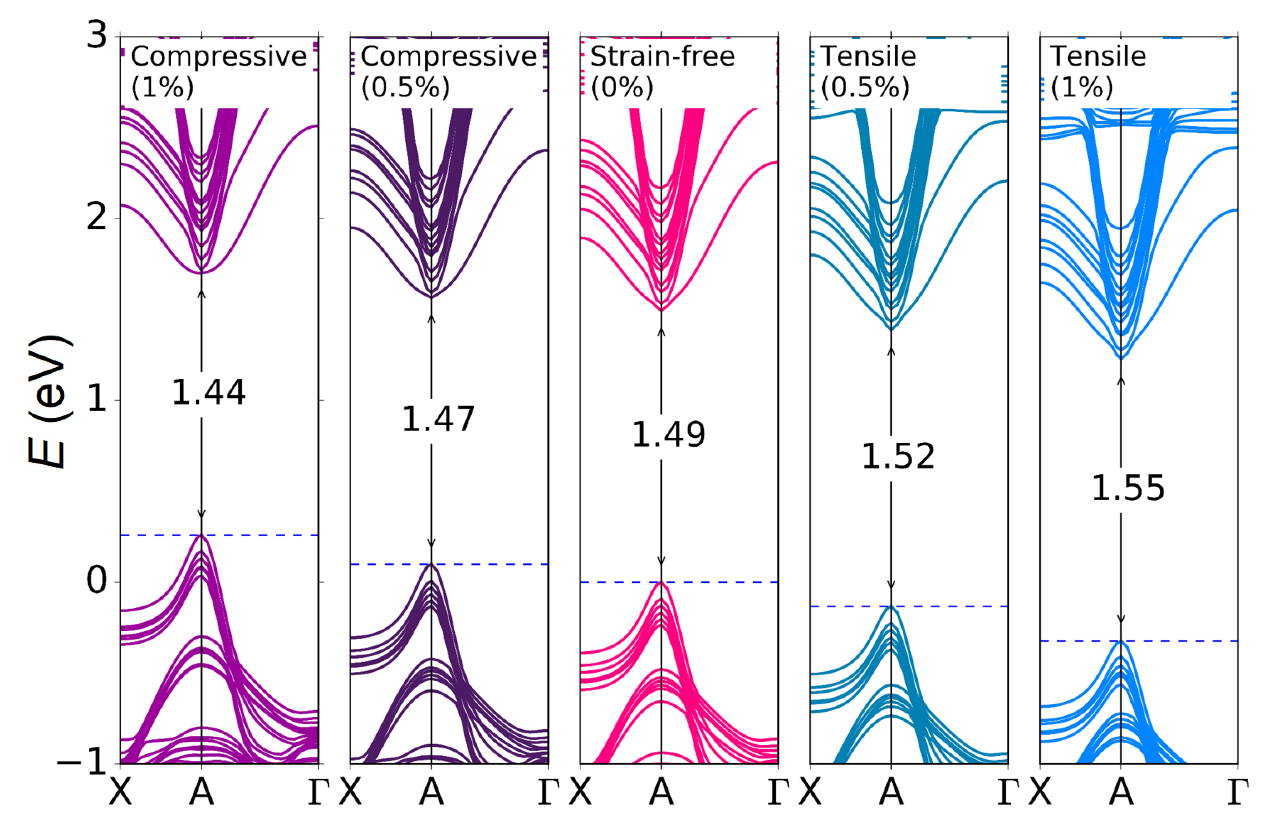


**Supplementary Figure 13 |** The band structure of different strain conditions from compressive state to tensile state and deformation variables are is 1%, 0.5%, 0%, -0.5%, -1%, respectively.


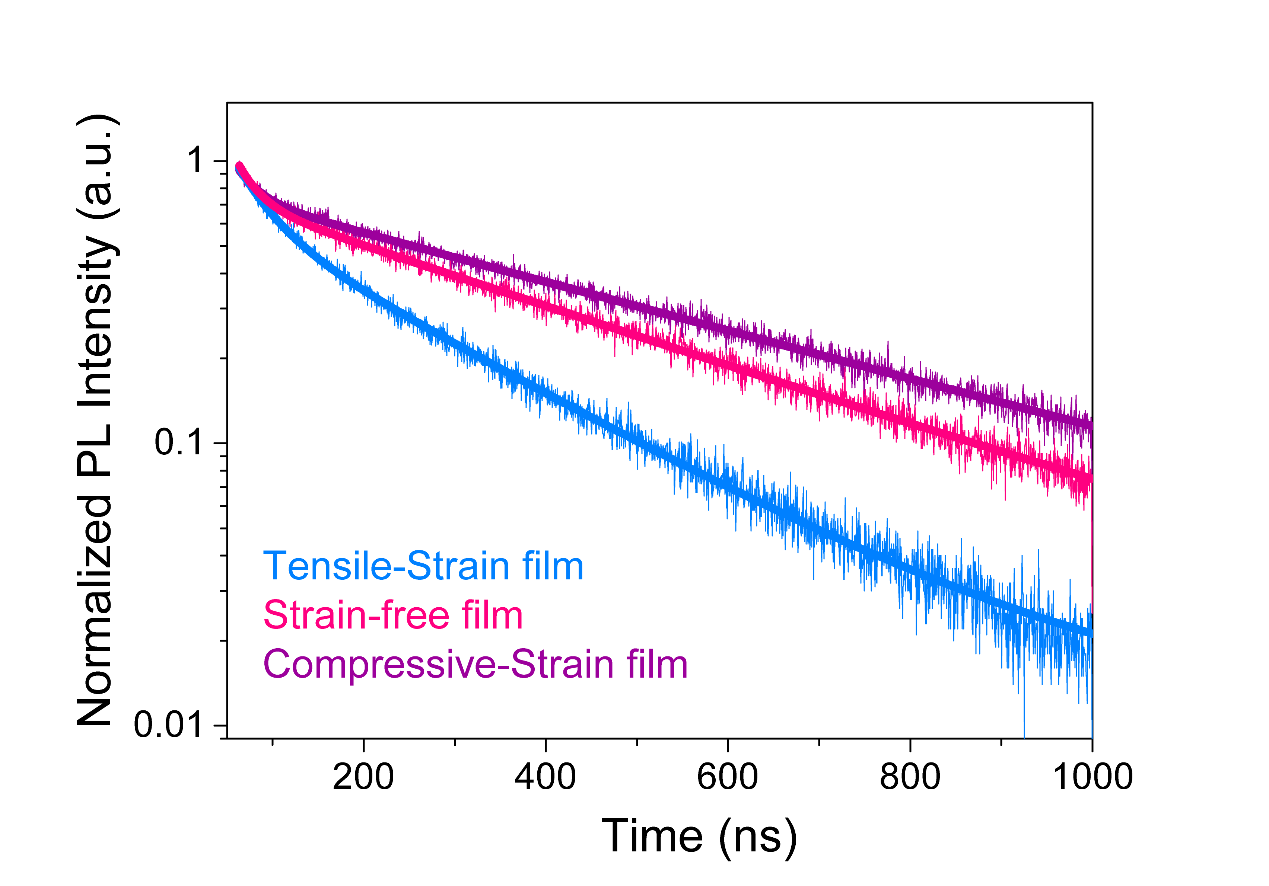


**Supplementary Figure 14** **|** The time-resolved photoluminescence spectra of perovskite films with tensile, strain-free and compressive state.


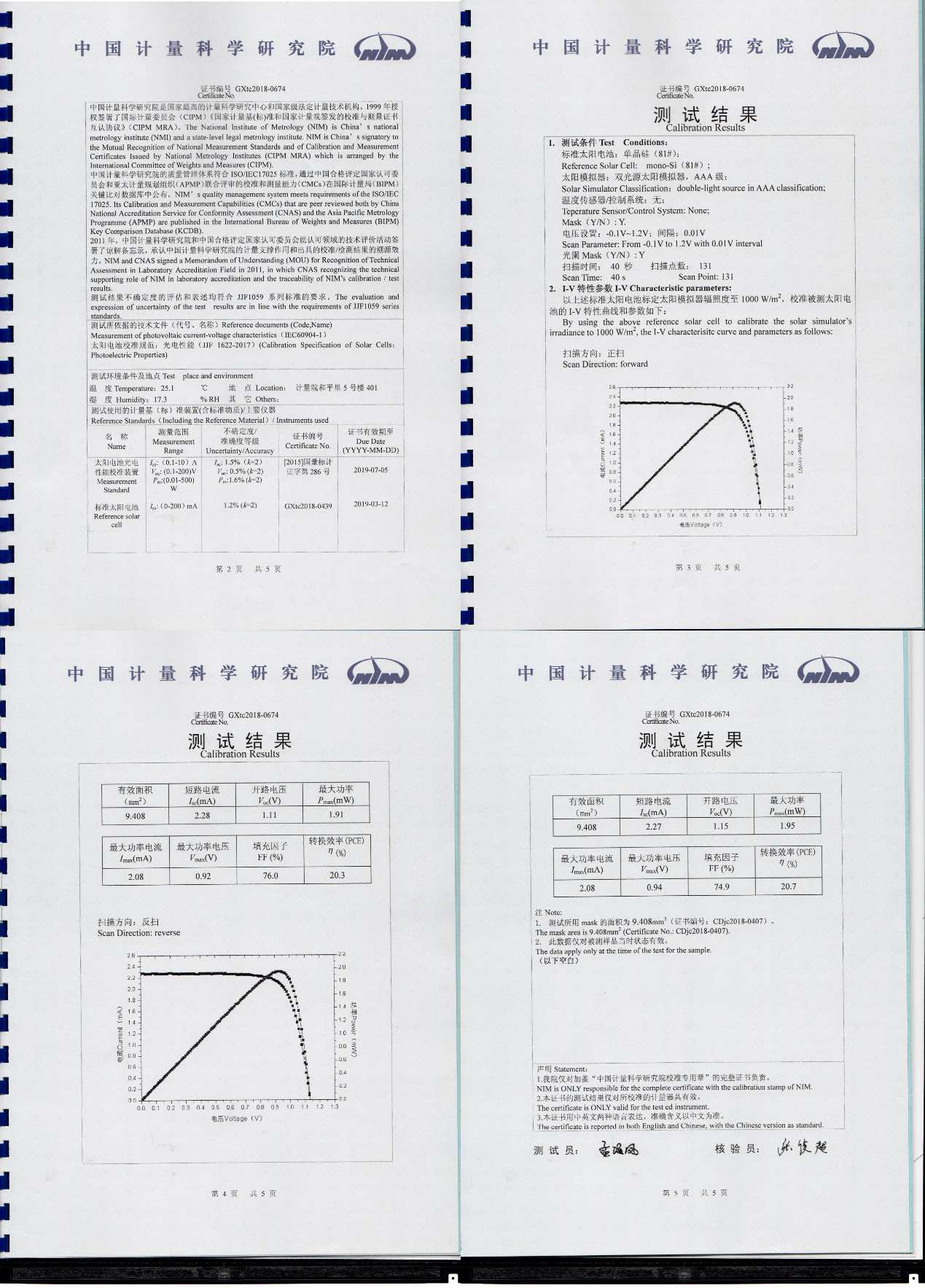


**Supplementary Figure 15** **|** Certificated results from an accredited photovoltaic certification laboratory (NIM, China). The certificated efficiency is **20.7%** in reverse scan and **20.3%** in normal scan direction. The mask area of **9.408 mm^2^** has been used.

**Supplementary Table 1:** Instrument angles (*ω, ψ, ϕ*) setting parameters of GIXRD Residual strain gradient measurement.

| *τ*[nm] | （hkl） | *ψ*[°] | *ω*[°] | *Φ* [°] |
| --- | --- | --- | --- | --- |
| 50 nm | (001) | 5 | 0.3153 | -36.63 |
|  |  | 50 | 0.4953 | -81.53 |
|  | (012) | 5 | 0.3114 | -17.45 |
|  |  | 50 | 0.4854 | -70.32 |
|  | (022) | 5 | 0.3107 | -13.47 |
|  |  | 50 | 0.4838 | -64.81 |
| 200 nm | (001) | 5 | 2.011 | -44.96 |
|  |  | 50 | 3.594 | -85.56 |
|  | (012) | 5 | 1.831 | -19.31 |
|  |  | 50 | 2.954 | -73.42 |
|  | (022) | 5 | 1.807 | -14.61 |
|  |  | 50 | 2.887 | -67.74 |
| 500 nm | (001) | 5 | 4.599 | -64.31 |
|  |  | 50 | 7.000 | 90.00 |
|  | (012) | 5 | 3.480 | -21.76 |
|  |  | 50 | 5.924 | -77.20 |
|  | (022) | 5 | 3.384 | -15.99 |
|  |  | 50 | 5.596 | -71.09 |

**Supplementary Table 2:** The statistics of various averaged lattice distance at the depth of 50 nm, 200 nm, 500 nm for the (222) (004) (113) crystal plane family from TEM diffraction data, related to tensile-strain film.

| Depth | (222) | (004) | (113) |
| --- | --- | --- | --- |
| 50 nm | 1.75 ± 0.04 Å | 1.62 ± 0.02 Å | 1.96 ± 0.03 Å |
| 200 nm | 1.85 ± 0.03 Å | 1.65 ± 0.01 Å | 2.01 ± 0.02 Å |
| 500 nm | 1.89 ± 0.04 Å | 1.67 ± 0.02 Å | - 1. ± 0.01 Å |

**Supplementary Table 3:** The slope of residual strain fitting line at the depth of 50 nm, 200 nm, 500 nm for the tensile-strain film and strain-free film.

| Depth | Strain type | Slope(%)  (001) | Slope(%)  (012) | Slope(%)  (022) |
| --- | --- | --- | --- | --- |
| 50nm | Tensile-strain | -5.0 | -6.1 | -24.7 |
|  | Strain-free | -0.8 | -1.6 | -7.23 |
| 200nm | Tensile-strain | -2.6 | -2.2 | -9.81 |
|  | Strain-free | -0.5 | -0.8 | -2.9 |
| 500nm | Tensile-strain | -0.8 | -0.9 | -2.31 |
|  | Strain-free | -0.2 | 0.0 | -0.3 |

**Supplementary Table 4:** Average photovoltaic parameters of various solar cells fabricated with different flipped annealing time.

| Device | *V*oc (V) | *J*sc (mA/cm^2^) | FF (%) | PCE (%) |
| --- | --- | --- | --- | --- |
| Reference | 1.07 ± 0.04 | 22.23 ± 0.56 | 71.86 ± 3.72 | 17.29 ± 1.54 |
| 15min | 1.10 ± 0.02 | 22.32 ± 0.62 | 72.92 ± 2.98 | 17.96 ± 1.41 |
| 25min | 1.13 ± 0.02 | 22.46 ± 0.63 | 74.55 ± 2.42 | 19.25 ± 0.96 |
| 35min | 1.12 ± 0.03 | 21.60 ± 0.39 | 75.44 ± 4.43 | 18.06 ± 1.99 |
| 45min | 1.03 ± 0.01 | 19.80 ± 0.71 | 57.71 ± 3.90 | 11.75 ± 0.94 |

**Supplementary Table 5:** Fitted decay times from TPV/TPC spectra for mixed perovskite films with tensile-strain and strain-free state.

|  | Tensile-strain | Strain-free |
| --- | --- | --- |
| TPC (μs) | 12.96 | 1.06 |
| TPV (μs) | 4.01 | 10.32 |

**Supplementary Table 6:** Fitted parameters of TRPL curves for the tensile, strain–free, compressive perovskite films and glass/ perovskite film(tensile, strain–free, compressive) deposited on various substrates. Specific calculation method can be found in **Supplementary Notes.**

| Sample | A_1_ | τ_1_ (ns) | A_2_ | τ_2_(ns) | τ_avg_(ns) |
| --- | --- | --- | --- | --- | --- |
| Tensile  PVSK | 0.77 | 234.6 | 1.87 | 37.5 | 179.5 |
| Strain–free PVSK | 0.81 | 395.3 | 3.80 | 23.6 | 314.5 |
| Compressive  PVSK | 2.70 | 24.1 | 0.83 | 491.3 | 426.6 |
| Tensile PVSK/HTM | 57.66 | 3.9 | 0.48 | 77.99 | 14.6 |
| Strain–free PVSK/HTM | 61.73 | 4.8 | 0.0063 | 73.2 | 4.9 |

**Supplementary notes**

The PL decay time was fitted by using a bi-exponential equation

$$f\left( t \right)=A_{1}\timesⅇ^{\frac{-t}{\tau_{1}}}+A_{2}\timesⅇ^{\frac{-t}{\tau_{2}}}$$

where A1 and A2 are the relative amplitude fraction for each decay component and τ1 and τ_2_ are the fast and slow decay PL species and B is a constant.1,2 The average PL

lifetime is determined by the equation

$$\tau_{\mathrm{avg}}=\frac{A_{1}\tau_{1}^{2}+A_{2}\tau_{2}^{2}}{A_{1}\tau_{1}+A_{2}\tau_{2}}$$

**Supplementary methods**

**GIXRD Residual strain gradient measurement.** The macroscopic residual strain is an internal strain in polycrystalline materials that is balanced over a wide range of grains. X-ray diffraction (XRD) represents a common technique to evaluate residual stress/strain gradients along the surface normal direction in polycrystalline thin films and coatings from measured X-ray elastic strains using the sin^2^*ψ* technique. The measured lattice spacing d and X-ray elastic strains ε represent volume-average quantities which depend on the actual stress/strain depth profile, X-ray penetration depth, reflection plane (hkl) and experiment geometry. In general, d and ε can be related by$\varepsilon=\left( d-d_{0} \right)/d_{0}$. According to the Braggs law, Hooke's law and equations of equilibrium, the classic sin^2^ψ equation about stress *σ* and 2*θ* can be obtained as follow

$\sigma=-\frac{E}{2\left( 1+\nu\right)}\frac{\pi}{180}\cot\theta_{0}\frac{\partial\left( 2\theta\right)}{\partial\sin^{2} \psi}$ (1)

where *E* and υ are Young’s modulus and Poisson’s ratio of the thin film, respectively. *θ_0_* is the diffraction peak for stress free perovskite (hkl) crystal plane and *θ* is the diffraction peak for the actual perovskite thin films. The *ψ* is the angle the diffraction vector with respect to the sample normal direction.

Here we define

$$C=-\frac{E}{2\left( 1+\nu\right)}\frac{\pi}{180}\cot\theta_{0} k^{'}=\frac{\partial\left( 2\theta\right)}{\partial\sin^{2} \psi}$$

We can use C and $k^{'}$ to simplify the formula. When determining the diffraction angle, C is a constant and there is a linear function between 2θ and sin^2^ψ, $k^{'}$ is the slope. Then, we can transfer the formula (1) to

$\varepsilon=\frac{\sigma}{E}=\frac{c}{E}k^{'}$ (2)

According to the formula (2), the state of the residual stress σ and macroscopic residual strain ε can be judged by the slope of 2*θ*-sin^2^*ψ* line. When $k^{'}<0$, the ε value is positive and it is tensile strain/stress, $k^{'}>0$, the ε value is negative then it is compressive strain/stress, the magnitude of strain/stress is determined by the value of the slope.

In this work, the perovskite film samples are considered to be quasi-isotropic. Due to the high X-ray absorbance of lead halide perovskites, the depth-resolved X-ray residual strain characterization can provide reliable depth resolution and fine structural information. To perform the GIXRD residual strain gradient measurement, the Braggs reflection have to be scanned at various values by tilting the samples around the axis so that we can evaluate (hkl, *τ*, *ψ*) and diffraction depth dependencies experimentally in the Laplace space. It must be emphasized here that the instrument angle and diffraction geometry are no longer equivalent when the strain is tested using grazing incident method. To describe the relationship of the instrumental angles (*ω*, *ψ*, *ϕ*, *θ*) and diffraction geometry angles (*α*, *β*, *φ*, *δ*), instrument coordinate system（Z_1_,Z_2_,Z_3_）and sample reference system(S_1_,S_2_,S_3_) were introduced in **Supplementary Figure 1**. The instrument setting parameters is fixed while the sample reference rotates around the axis Z_2_(the instrumental angle ψ) and the axis Z_1_(the instrumental angle ω). The instrumental angle ϕ is the rotation angle around the normal of the sample surface. Diffraction geometry angles (*α*, *β*, *φ*, *δ*) in the Laplace space was present in **Supplementary Figure 1**. The inclination of the diffraction vector with respect to the sample normal is given by the angle φ in diffraction geometry. Incident angle α and diffracted angles β together determine the X-ray penetration depth and all diffraction geometry angles can be replaced by the instrumental angles^1, 2, 3, 4^

The relationships between the diffraction geometry angles (φ, δ) and the instrumental angles (ω, ψ, ϕ, θ) are described with the formulas as follows^1, 2, 3, 4^

$\cos\varphi=\cos\psi\cos\left( \omega-\theta\right)$ (3)

$\delta=\phi+\mathrm{arc}\tan\left[ -\sin\psi/\tan\left( \omega-\theta\right) \right]$ (4)

Taking into account about 600nm thick perovskite film, the information depth τ_t_, is determined with the instrumental angles (*ω*, *ψ*, *θ*), linear absorption coefficient μ and the sample thickness *t* in this study: Delhez et al

$\tau_{t}=\frac{1}{k\mu}+\frac{t}{1-\frac{1}{\exp\left( -\mu kt \right)}}$ (5)

With $k=\frac{2\sin\theta\cos\left( \theta-\omega\right)}{\cos\psi\left[ \sin^{2} \theta-\sin^{2} \left( \theta-\omega\right) \right]}$

*μ* is the linear absorption coefficient of the irradiated material.

When determining the crystal face(2*θ* is determined), a series of instrumental angles ψ are proposed for a defined angular zone, the series of incident angles ω can be calculated from equation (5) for a selected depth *τ*. Thus, the polar angles φ can be obtained from equation (3) according to the calculated *ω* value. In equation (4), corresponding instrumental angle *ϕ* was calculated to make the angle *φ* remain constant.

All perovskite films were characterized using a Rigaku SmartLab five-axis X-ray diffractometer equipped with Cu K radiation at 45 kV and 200 mA, parallel beam optics and a secondary graphite monochromator. Before the test, the X-ray diffraction on well recrystallized LaB_6_ powders was used for subtle alignment of instrument, the acceptable LaB_6_ peak shift is less than 0.01° in 2*θ* comparing to its JCPDF file. Simply, we fixed the 2*θ* and varied the instrument tilt angle *ψ* to obtain corresponding XRD patterns. At each depth (from 50 nm to 500 nm), a series of instrumental angles (*ω*, *ψ*, *ϕ*) was individually proposed for experimental peak acquisitions and strain analysis. The measurements were conducted through different instrument tilt angles from 0°to 50° with the depth of 50 nm, 200 nm, 500 nm, respectively. The corresponding test parameters (*ω*, *ϕ*) are shown in the table S1. The (001), (012), and (022) plane of mixed perovskite was scanned with a 0.02° min^-1^ to ensure fine structural information.

**Supplementary Reference**

1. Stefenelli M, et al. X-ray analysis of residual stress gradients in TiN coatings by a Laplace space approach and cross-sectional nanodiffraction: a critical comparison. *J. Appl. Crystallogr.* **46**, 1378-1385 (2013).

2. Noyan IC, et al. Residual stress/strain analysis in thin films by X-ray diffraction. *Crit. Rev. Solid. State Mater. Sci.* **20**, 125-177 (1995).

3. Benediktovitch A, et al. Sample tilt-free characterization of residual stress gradients in thin coatings using an in-plane arm-equipped laboratory X-ray diffractometer. *J. Appl. Crystallog.r* **47**, 1931–1938 (2014).

4. Chen Z, et al. Residual stress gradient analysis with GIXRD on ZrO 2 thin films deposited by MOCVD. *Surf. Coat. Technol.* **206**, 405-410 (2011).
